# A panoramic view of the molecular epidemiology, evolution, and cross-species transmission of rosaviruses

**DOI:** 10.1186/s13567-024-01399-3

**Published:** 2024-11-08

**Authors:** Minyi Zhang, Shunchang Fan, Minyi Liang, Ruojun Wu, Jingli Tian, Juxian Xian, Xiaofeng Zhou, Qing Chen

**Affiliations:** 1 Department of Epidemiology, Guangdong Provincial Key Laboratory of Tropical Disease Research, School of Public Health, Southern Medical University, Guangzhou, 510515 China; 2 Department of Epidemiology and Infectious Disease Control, Longhua Centre for Disease Control and Prevention, Shenzhen, 518109 China

**Keywords:** Rosavirus, rodent, shrew, evolution, Cross-species transmission

## Abstract

*Rosavirus* is a newly discovered member of the family *Picornaviridae* that was initially detected in wild rodents and subsequently in children with diarrhoea. Nevertheless, there is a significant gap in our understanding of the geographical distribution, phylogenetic relationships, evolutionary patterns, and transmission of rosaviruses. To address these issues, we analysed 434 rodents and shrews from five different species that were collected in southern China. Using PCR screening of faecal samples, we detected rosaviruses in Norway rats (*Rattus norvegicus*) and identified two previously undocumented host species: tanezumi rats (*Rattus tanezumi*) and Asian house shrews (*Suncus murinus*). Rosaviruses were particularly common in these animals, with an overall prevalence rate of 32.49% (141/434). For genetic and evolutionary analyses, we selected six representative positive samples to amplify the complete genomes of rosaviruses. Bayesian phylogenetic analysis suggested that our sequences clustered within the genus *Rosavirus,* where genotype B sequences are the closest relatives. The elevated nonsynonymous-to-synonymous ratios observed in rosavirus B may be attributed to relaxed selection pressures driven by virus spillover events. On the basis of the available data, it is hypothesized that the genus *Rosavirus* may have originated from Norway rats around the year 1339. In summary, these findings provide valuable insights into the complex evolutionary history of rosaviruses and underscore the urgent need for ongoing surveillance of this virus.

**Supplementary Information:**

The online version contains supplementary material available at 10.1186/s13567-024-01399-3.

## Introduction

In recent years, the incidence of infectious diseases has increased, posing significant risks to both global security and public health. Notable examples include the epidemic of the Ebola virus, severe acute respiratory syndrome coronavirus (SARS-CoV), Middle East respiratory syndrome coronavirus (MERS-CoV), and SARS-CoV-2 [1, 2]. Most human epidemic viruses are zoonotic or originate in nonhuman animals, making pathogens prevalent in a variety of animals more likely to emerge in human populations through cross-species transmission [3, 4]. Accordingly, surveillance of the incidence and genetic characterization of potentially pathogenic viruses in animal populations are highly important for mitigating viral outbreaks.

*Rosavirus* has been identified as a recent member of the family *Picornaviridae*. In accordance with the latest report from the International Committee on Taxonomy of Viruses (ICTV), the family *Picornaviridae* currently encompasses 158 known species differentiated into 68 genera (ICTV, 2023). Many of these species are associated with diseases of significance in both humans and animals. The source for this information can be accessed at Picornavirus Home [5]. Rosaviruses are small, nonenveloped viruses with positive-sense single-stranded RNA genomes. Their genetic structure resembles that of other picornaviruses, consisting of VPg, 5′UTR^IRES-II^, a single polyprotein encoding the structural protein P1 (VP4-VP2-VP3-VP1) and non-structural proteins P2 (2A^H-box/NC^-2B-2C) and P3 (3A-3B^VPg^-3C^pro^-3D^pol^), a 3′UTR, and a poly (A) tail.

The name “rosavirus” originates from the identification of a picornavirus, designated M-7, initially found in a faecal sample from a canyon mouse (*Peromyscus crinitus*) in the United States of America (USA) in 2010 [6, 7]. A subsequent rosavirus, provisionally named rosavirus 2, was subsequently discovered in stools and was linked to cases of diarrhoea in children in Gambia [8]. Genomic analysis revealed that the rosavirus strain M-7 was the closest relative to rosavirus 2, and both strains were grouped into rosavirus A through a phylogenetic approach [8]. These findings indicate that rosaviruses have the potential for spillover from rodents to humans. There is consequently growing interest in elucidating the evolutionary history and assessing the cross-species transmission capacity of this virus.

In 2016, Lau et al. identified two additional species: rosavirus B from Norway rats (*Rattus norvegicus*) and rosavirus C from five different wild rodent species [greater bandicoot rat (*Bandicota indica*), coxing white-bellied rat (*Niviventer coxingi*), chestnut white-bellied rat (*Niviventer fulvescens*), Indochinese forest rat (*Rattus andamanensis*), and roof rat (*Rattus rattus*)] in Hong Kong, China [9]. These findings were linked to the development of multisystemic diseases in mouse models [9]. Similarly, a recent investigation in Hungary provided empirical support for the extraintestinal reproduction ability of rosavirus B (designated rat08/rRoB/HUN) [10]. These results suggest that rosaviruses retain the capacity to infect mammals and induce widespread illnesses within their hosts. Notably, rosaviruses can be among the pathogens that are routinely monitored in cases of diarrheal diseases. Previous studies have conducted molecular detection of rosaviruses in raw sewage samples and the stools of children, alongside other prevalent diarrheal viruses, such as rotavirus, norovirus, and astrovirus [11, 12].

Rodents represent the largest order of mammals globally, accounting for 43% of approximately 4800 living mammalian species [9]. Similarly, shrews, despite being small animals, have the potential to transmit pathogenic viruses to humans, particularly certain species, such as the Asian house shrew (*Suncus murinus*), which frequently inhabits areas in close proximity to human populations. The identification of hantaviruses in many shrew species has increased our understanding of the complex evolutionary dynamics involved in cross-species transmission. However, previous research has not identified any instances of rosaviruses in samples obtained from shrews. The close proximity of urban rats and shrews to humans and domestic animals increases the likelihood of cross-species transmission [13]. This transmission can occur through direct or indirect contact with the carcasses, urine, faeces, and parasites of these animals [13]. While significant progress has been made in understanding the tissue distribution, pathogenicity, and interspecies transmission of rosavirus C [9], critical aspects of rosavirus evolution and potential for emergence remain unclear. These knowledge gaps pose challenges for effectively preventing and controlling the spread of the virus during outbreaks.

This work examined the presence of rosaviruses in faecal samples collected from diverse species of rodents and shrews across several areas of China. By utilizing current data pertaining to rosaviruses, we ran a series of bioinformatics analyses to characterize their genetic diversity, evolution and transmission dynamics comprehensively.

## Materials and methods

### Sample collection

In total, 434 faecal samples were collected from rodents and shrews whose health status was undetermined. They were captured close to human residences via live traps between October 2015 and September 2017 in four areas of China: Guangzhou (longitude 113°27°E, latitude 23°13°N) in Guangdong Province, Yiyang (longitude 112°36°E, latitude 28°57°N) in Hunan Province, Xiamen (longitude 118°04°E, latitude 24°26°N) in Fujian Province, and Malipo (longitude 104°70°E, latitude 23°12°N) in Yunnan Province. The procedures for sampling and sample processing were reviewed and approved by the Animal Ethics and Welfare Committee of the School of Public Health, Southern Medical University, China. All mammals were captured alive and maintained in full adherence to the requirements outlined in the Rules for the Implementation of Laboratory Animal Medicine (1998) from the Ministry of Health, China. The classification of animal species was first performed by experienced field biologists on the basis of morphological characteristics and then verified through sequence analysis of the mitochondrial cytochrome b (*cytb*) gene from each sample. Individual fresh stool samples were promptly transferred into RNase-free tubes containing 700 µL of phosphate-buffered saline (PBS) with a 0.3% homogenate and stored at −80 ℃ before further processing.

### Nucleic acid extraction and reverse transcription

The thawed faecal samples were fully resuspended in PBS and centrifuged at 10000 × *g* for 10 min. Viral nucleic acid was extracted from 200 µL of each supernatant via a MiniBEST Viral RNA/DNA Extraction Kit (TaKaRa, Japan). The cDNA was subsequently reverse transcribed into synthesized cDNA via a Transcriptor First Strand cDNA Synthesis Kit (Roche, Switzerland) and random hexamer primers following the manufacturer’s instructions. The synthetic cDNA was either utilized directly as the template for PCR or kept frozen at −20 °C.

### Detection of rosavirus

The presence of rosavirus was detected by using an established PCR protocol based on a specific primer pair designed for the conserved 3D RNA-dependent RNA polymerase (3D^RdRp^) gene (600 bp), in line with prior research [10]. The PCR mixture for each sample had a total volume of 25 µL, including 12.5 µL of Green Master Mix (Promega, USA), 8.5 µL of sterilized H_2_O, 2 µL of cDNA template, and 1.0 µM of each primer. The mixtures were amplified according to the following protocol: an initial denaturation step at 95 ℃ for 2 min, followed by 40 cycles of denaturation at 95 ℃ for 30 s, annealing at 56 ℃ for 1 min, and extension at 72 ℃ for 1 min, together with a final extension at 72 ℃ for 5 min. Amplicons were subjected to 1.2% agarose gel electrophoresis and sequenced using an ABI Prism 3730xl DNA Analyzer (Applied Biosystems, Foster City, CA, USA).

### Acquisition of complete polyprotein sequences

Among the rosavirus-positive samples, we selected representative samples to obtain complete polyprotein sequences. The selection was based on a combination of criteria, including representative geographic distribution, host species, genetic diversity, and high-quality RNA samples. Fifteen pairs of primers were designed to amplify the complete polyprotein sequences of rosavirus using the Benchling website [14], based on the Hungarian sequence rat08/rRoB/HUN (accession no. MN116648). The primer sequences are shown in Additional file 1. After sequencing, contigs with overlaps were assembled using the SeqMan program implemented in Lasergene software (DNASTAR, Inc. Madison, WI, USA).

### Genome and phylogenetic analyses

The nucleotide (nt) sequences of rosavirus identified in the present study were compared with the corresponding sequences of other rosavirus sequences available in GenBank using Basic Local Alignment Search Tool (BLAST) provided by the National Center for Biotechnology Information (NCBI) [15]. Multiple sequence alignment was accomplished using MAFFT algorithm [16], which involves corresponding segments of rosavirus reference sequences in GenBank. The putative amino acid (aa) of the open reading frame (ORF) was determined by employing the ORF finder tool with default parameters [17] for the identified sequences. The calculation of pairwise nt and aa identities was performed using BioAider v1.527 [18]. Phylogenetic analyses were generated using the Bayesian technique implemented in MrBayes v3.2 [19]. The most appropriate model of nt or aa substitution for each dataset was established using ModelFinder v1.6.8 based on the Bayesian information criterion [20]. The plotting, annotation, and visualization of the trees were achieved using *ggtree* package in R software v4.2.3 [21–23]. GenBank accession number, host species, geographical regions, and collection dates for all the rosavirus sequences included in the present study are listed in Additional file 2.

### Coevolutionary analysis

Host species were collected, and their corresponding sequences targeting the complete *cytb* gene were retrieved from GenBank. The full-length P1 region of rosaviruses was utilized for coevolutionary analysis. Maximum likelihood phylogenetic trees were inferred via RaxML v8.1.17. A distance-based global-fit approach was employed to assess the congruence between viral and host topologies on phylogenetic trees in ParaFit [24]. The event-based coevolutionary findings were generated by processing the phylogenetic trees of both the virus and the host using Jane v4.0 [25]. We assigned event costs = 1 for duplications, host-switching, virus loss, and failure to diverge following host cospeciation, whereas codivergence events were assigned a cost of 0. The number of generations and population size were both set at 100. The cophylogenetic tree, which visually represents the connections between each virus and its corresponding host species, was constructed via the “cophylo” function of the *phytools* package in R [26].

### Recombination and mutation analyses

Using the RDP, GENECONV, bootscan, maximum chi square, Chimera, SISCAN, and 3SEQ scanning methods, the Recombination Detection Program v3 (RDP3) was utilized to recognize potential recombination events in complete polyprotein sequences of rosaviruses [27]. All analyses were performed with a Bonferroni correction and the highest acceptable *p* value = 0.05 to minimize the possibility of Type I errors. Recombination events detected by more than three methods were considered reliable and were embedded in all the genomes. We subsequently used Simplot v3.5.1 to generate a similarity plot to validate the recombination breakpoints and visually inspect the similarities between our sequences and other relevant reference sequences. A substitution mutation analysis of rosaviruses was carried out via BioAider v1.527 [18] to identify and characterize changes in the viral genomes by comparison with the earliest reported reference sequence (accession no. JF973686). Five distinct mutation types, namely, synonymous, nonsynonymous, insertion, deletion, and termination, were examined via a codon-based methodology.

### Selection pressure analyses

To perform a comparative analysis of the selection pressures acting on rosavirus B and rosavirus C, all currently available datasets were compiled for ten proteins (VP4, VP2, VP3, VP1, 2A, 2B, 2C, 3A, 3B, 3C, and 3D). The analysis could not be performed for rosavirus A (n = 2) because of the necessity of including a minimum of three distinct sequences for the selection techniques to be applicable. The nonsynonymous to synonymous (*dN/dS*) rate ratios were estimated using the single-likelihood ancestor counting (SLAC) approach [28] on the Datamonkey Webserver [29, 30]. Next, the *dN/dS* ratios of the internal and external branches were compared using both the two-ratio model and the one-ratio model using CODEML available in PAML [31]. The likelihood ratio test (LRT) was implemented to assess the presence of positive selection pressure on the internal branch. Additionally, the mixed effects model of evolution (MEME) approach in Datamonkey and the Bayes empirical Bayes (M8 model + BEB) method [32] in PAML were utilized to identify positively selected sites for each gene. All the statistical tests were deemed statistically significant at a significance level of *P* < 0.05.

### Time-scale evolutionary characterization

All available partial capsid coding P1 gene (VP1) sequences of rosaviruses from GenBank, along with the six samples amplified in this study, were obtained and aligned using the MAFFT with the L-INS-i strategy [16]. The time-scale phylogeny of rosavirus was constructed using the Bayesian Monte Carlo Markov chain (MCMC) method as implemented in BEAST v1.10.4 [33]. A general time-reversible (GTR) model with a gamma distribution (G) across sites was determined as the best-fitting substitution model using ModelFinder [20]. We compared various molecular clock models and coalescent models using path sampling to estimate marginal likelihoods. The findings revealed that the optimal tree prior for our dataset was the combination of a strict clock and Bayesian skyline coalescent [34, 35], as shown in additional file 3. On the basis of recent data regarding the evolution rate of the VP1 gene in closely related picornaviruses, we adopted an informative prior for the substitution rate parameter, using a normal distribution (mean rate = 2 × 10^–3^ substitutions/site/year with SD = 5 × 10^–4^) [36, 37]. The MCMC chains were run for 100 million iterations with sampling every 10 000 states and 10% burn-in. Estimates were made for the mean substitution rate and the time to the most recent common ancestor (tMRCA), along with the highest posterior density (HPD) regions at a 95% confidence level. MCMC convergence and the effective sample size (ESS) of all estimated parameters were assessed using Tracer v1.7 [38]. The ultimate Bayesian maximum clade credibility (MCC) tree was produced using the TreeAnnotator program v2.6.3 and subsequently visualized in Figtree software v1.4.4.

### Discrete phylogeography analysis

Simultaneously, phylogeographic analysis was conducted using BEAST to reconstruct the geographical distribution, diffusion rates, migration patterns, and zoonotic origin, providing a comprehensive understanding of the viral dissemination network. Seven geographical locations (USA, Gambia, Hungary, Hong Kong, Guangzhou, Yiyang, and Fujian) and ten host species (human, Norway rat, canyon mouse, Indochinese forest rat, chestnut white-bellied rat, roof rat, coxing white-bellied rat, lesser ricefield rat, tanezumi rat, and Asian house shrew) were treated as discrete states and hosts, respectively. The asymmetric substitution model with Bayesian stochastic search variable selection (BSSVS) was applied in BEAST to infer statistically significant diffusion rates between geographic areas and host species. Diffusion rates between discrete traits were calculated using SPREAD3 package, and the generated log files were then used to determine the Bayes factor (BF) among discrete locations and host species. Significant migration pathways were identified using both a BF > 3 and a posterior probability > 0.5.

## Results

### Prevalence of rosavirus in rodents and shrews

Using PCR targeting the partial 3D^RdRp^ of rosavirus, 434 faecal samples of rodents and shrews collected from Guangzhou (*n* = 213), Yiyang (*n* = 108), Xiamen (*n* = 61), and Malipo (*n* = 52) in southern China were screened for the presence of rosavirus. Overall, 32.49% (141/434) of the samples tested positive for rosavirus RNA. The prevalence was highest in Norway rats at 48.25% (124/257), followed by 45.45% (15/33) in tanezumi rats (*Rattus tanezumi*) and 1.77% (2/113) in Asian house shrews, as indicated in Table 1. Notably, rosavirus was not detected in either the lesser ricefield rat (*Rattus losea*) or the greater bandicoot rat (*Bandicota indica*) in this study.

**Table 1:** **Prevalence of rosavirus in rodents and shrews from southern China**

Scientific name	Common name	Guangzhou	Yiyang	Xiamen	Malipo	Total (%)
*Rattus norvegicus*	Norway rat	51/105	54/89	2/12	17/51	124/257 (48.25)
*Rattus tanezumi*	Tanezumi rat	2/3	13/19	0/10	0/1	15/33 (45.45)
*Rattus losea*	Lesser ricefield rat	–	–	0/30	–	0/30
*Bandicota indica*	Greater bandicoot rat	–	–	0/1	–	0/1
*Suncus murinus*	Asian house shrew	2/105	–	0/8	–	2/113 (1.77)
Total (%)		54/213 (25.35)	67/108 (62.04)	2/61 (3.28)	17/52 (32.69)	141/434 (32.49)

Rosavirus RNA-positive specimens/total specimens; “–” no animals were captured.

### Genomic characterization

To elucidate the genetic characteristics and diversity of rosaviruses, complete or nearly complete genome sequences of the identified rosaviruses were obtained through the utilization of fifteen primer pairs. Among these, three sequences derived from Norway rats were provisionally designated YY2 (accession no. PQ045667), YY4 (PQ045668), and YY6 (PQ045669), with genome sizes ranging from 7878 to 7925 nt. Two sequences, temporarily named YY10 (PQ045670) and YY27 (PQ045671), emerged in tanezumi rats with genome sizes of 7875 nt and 7880 nt, respectively. Another sequence, denoted SMU442 (PQ045672), was identified in an Asian house shrew and had a length of 7875 nt. These sequences presented a G + C content ranging from 50.72 to 51.14% and contained a single complete ORF encoding a putative polyprotein between 2491 and 2506 aa.

The genomic organization of our sequences was similar to that of previously known rosaviruses and featured a typical gene order of 5’UTR, viral capsid proteins (P1), nonstructural proteins (P2 and P3), and 3′UTR-poly (A). The hypothesized protease cleavage sites of our sequences were consistent with those of known rosaviruses, except for a projected cleavage site of E/S located between VP3 and VP1 in SMU442 (Table 2). Additionally, the protease-cleavage sites located between 3A and 3B (E/G) and 3B and 3C (E/G), together with 3C and 3D (G/L), were highly conserved with those typically found throughout the genus.

**Table 2:** **Protease cleavage sites of the rosavirus sequences in this study and other** ***Rosavirus* reference sequences from different host species**

Sequence	Cleavage site in										
	VP4/VP2	VP2/VP3	VP3/VP1	VP1/2A	2A/2B	2B/2C	2C/3A	3A/3B	3B/3C	3C/3D	VP4/VP2
YY2|*Rattus norvegicus*	LN/SS	FQ/ND	KE/GV	PQ/YG	NW/IP	QE/SP	FE/NP	EE/GA	YE/GL	PG/LK	LN/SS
YY4|*Rattus norvegicus*	LN/SS	FQ/ND	KE/GV	PQ/YG	NW/IP	QE/SP	YE/NP	EE/GA	YE/GL	PG/LK	LN/SS
YY6|*Rattus norvegicus*	LN/SS	FQ/ND	KE/GV	PQ/YG	NW/IP	QE/SP	FE/NP	EE/GA	YE/GL	PG/LK	LN/SS
YY10|*Rattus tanezumi*	LN/SS	FQ/ND	KE/GV	PQ/YG	NW/IP	QE/SP	FE/NP	EE/GA	YE/GL	PG/LK	LN/SS
YY27|*Rattus tanezumi*	LN/SS	FQ/ND	KE/GV	PQ/YG	NW/IP	QE/SP	FE/NP	EE/GA	YE/GL	PG/LK	LN/SS
SMU442|*Suncus murinus*	LN/SS	FQ/ND	KE/SV	PQ/YG	NW/IP	QE/SP	FE/NP	EE/GA	YE/GL	PG/LK	LN/SS
JF973686|*Peromyscus crinitus*	LN/DS	YE/SP	KE/HV	PE/LG	HW/LP	QE/AP	YE/AP	EE/GA	YE/GL	AG/LP	LN/DS
KJ158169|*Homo sapiens*	LN/SS	YE/SP	KE/HV	PQ/LG	HW/IP	QE/SP	YE/AP	EE/GA	YE/GL	TG/LP	LN/SS
KX783423|*Rattus norvegicus*	LN/SS	FQ/ND	KE/GV	PQ/YG	NW/IP	QE/SP	FE/NP	EE/GA	YE/GL	PG/LK	LN/SS
KX783425|*Rattus andamanensis*	LN/DS	PE/GD	EQ/SP	PQ/LG	HW/TK	ME/KG	FE/NS	AE/GA	FE/GL	KG/LR	LN/DS
KX783427|*Niviventer fulvescens*	LN/DS	PE/GD	EQ/AP	PQ/LS	HW/TK	ME/KG	FE/NS	TE/GA	FE/GL	EG/LR	LN/DS
KX783430|*Rattus rattus*	LN/NS	AE/GD	EQ/AP	PQ/MS	HW/TK	ME/KG	FE/NS	AE/GA	FE/GL	KG/LR	LN/NS
KX783432|*Niviventer coxingi*	LN/DS	LE/GD	EQ/VP	PQ/LS	HW/TR	LE/KG	FE/NS	SE/GA	FE/GL	KG/LK	LN/DS
KX156156|*Rattus losea*	LN/DS	PE/GD	EQ/VP	PQ/LS	HW/TK	ME/KG	FE/NS	AE/GA	FE/GL	KG/LK	LN/DS

**Figure 1:**
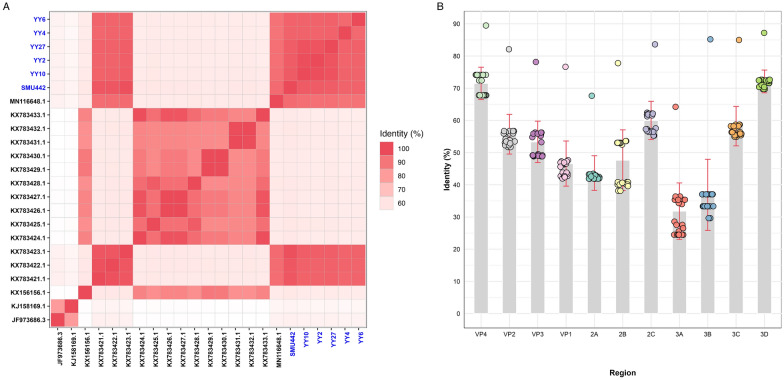
**Pairwise amino acid identity of complete polyprotein sequences A and mean pairwise amino acid identity of each coding region B of rosaviruses (%).** The sequences identified in this study are marked with a blue font.

Multiple sequence alignment revealed that our sequences isolated from rodents and shrews shared 88.3–98.1% nt and 93.2–99.0% aa identities with one another. A comparative analysis indicated that these sequences exhibited the closest relatedness to reference sequences found in Norway rats but shared less than 60% aa identity with rosavirus strains in humans and other rodent species (Figure 1A). The findings of mean pairwise identity among various coding regions indicated that 3A had greater genetic diversity than the other segments did at the aa level (Figure 1B). Compared with the other proteins, the P1 capsid protein, particularly the VP1 protein, exhibited greater diversity (ANOVA test, *P* < 0.001; Additional file 4). As expected, the highly conserved 3D protein revealed a lower degree of diversity among the nonstructural proteins (ANOVA test, *P* < 0.001; Additional file 4).

### Phylogenetic analyses

**Figure 2:**
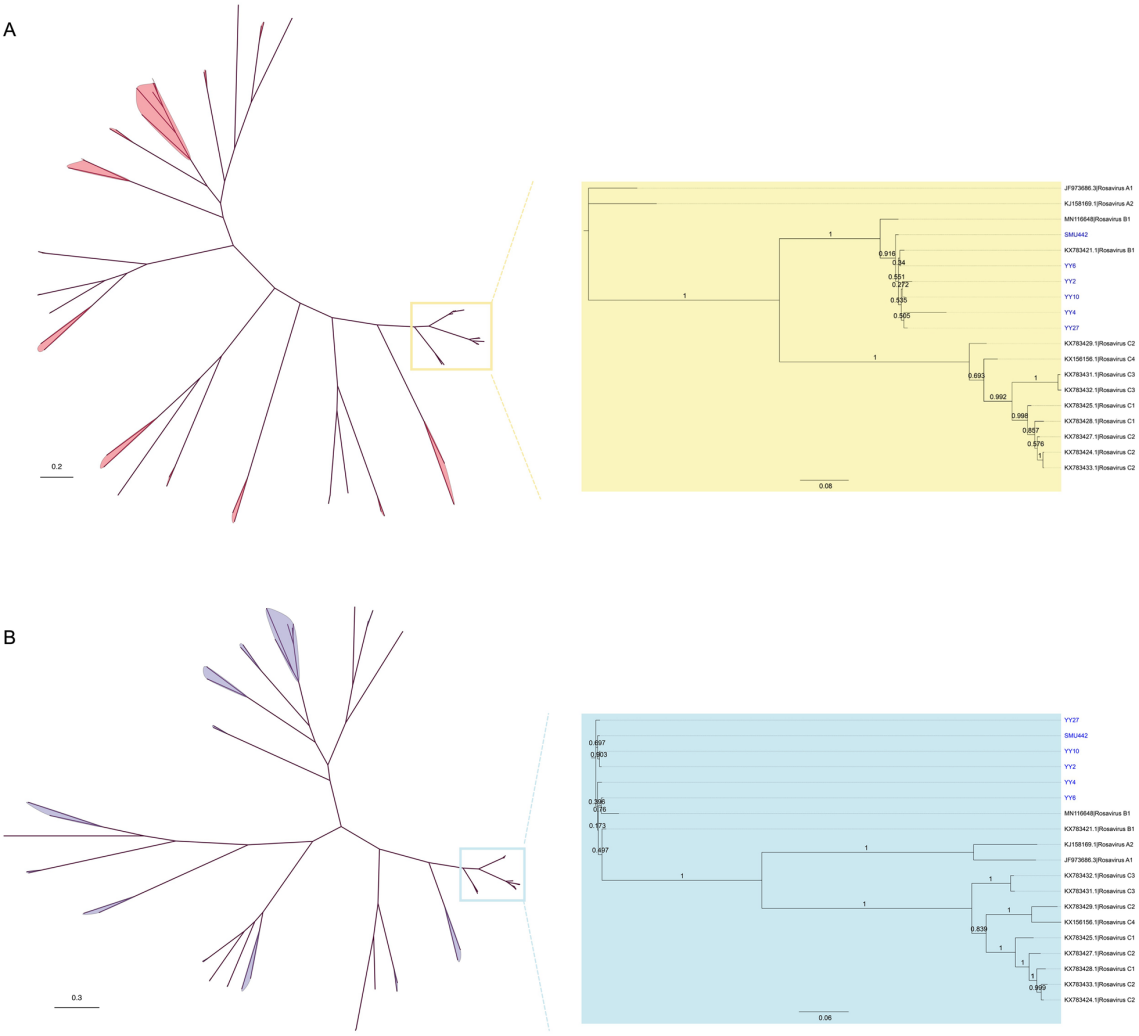
**Phylogenetic relationships of the members of the family** ***Picornaviridae***. Phylogenetic trees based on 2C (**A**) and 3CD (**B**) aa sequences were generated via the Bayesian method implemented in MrBayes. The numbers on each branch represent the posterior probability. The sequences identified in this study are marked with a blue font.

To elucidate the evolutionary relationships between the rosaviruses identified in this study and other members of the *Picornaviridae* family, we inferred Bayesian phylogenetic analyses on the basis of the amino acid (aa) sequences of the 2C and 3CD proteins, which are used to identify picornavirus members of a species. The phylogenetic trees based on the 2C and 3CD segments exhibited identical clustering patterns, indicating that the viruses identified here formed a distinct cluster within the genus *Rosavirus,* where the sequences of the rosavirus B species are the closest relatives (Figure 2).

**Figure 3:**
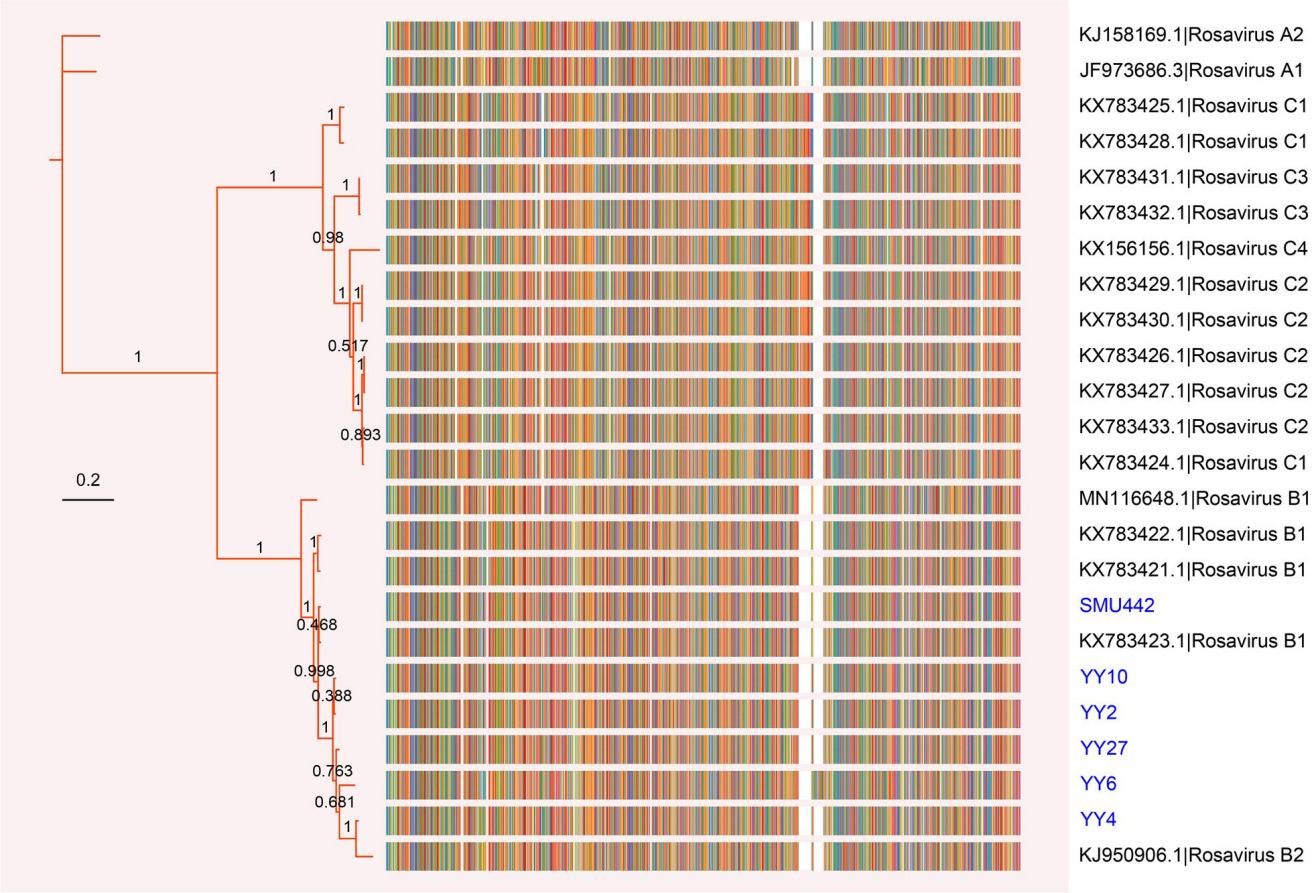
**Phylogenetic analysis of rosaviruses on the basis of P1 aa sequences**. A phylogenetic tree was constructed using the Bayesian method implemented in MrBayes. The visualization of the tree with a multiple sequence alignment was depicted by using the *ggtree* package in R. The numbers on branches represent posterior probabilities. The sequences identified in this study are labelled in blue font.

An evolutionary tree incorporating genome alignment visualization was subsequently constructed on the basis of complete aa sequences of the rosavirus P1 gene. All currently accessible P1 genes of rosaviruses can be categorized into three major phylogenetic clades according to their genotypes (Figure 3): (1) the rosavirus A clade, which encompasses a single rodent-borne rosavirus originating from the USA and another from a child with diarrhoea in Gambia; (2) the rosavirus B clade, which includes rodent-borne rosaviruses from the USA, China, and Hungary, as well as a virus carried by a previously unidentified host order, the Eulipotyphla; and (3) the rosavirus C clade, comprising rodent-borne rosaviruses identified in multiple host species from Hong Kong, China. Notably, the rodent-derived rosaviruses were distributed across all three clades, demonstrating considerable diversity. In this context, the viruses identified in this study grouped with sequences previously reported from Norway rats located at the same branch as rosavirus B. Specifically, our sequence YY4 showed a particularly close relationship with the rosavirus sequence NrRV/NYC-A15 (accession no. KJ950906), which was previously isolated from a Norway rat in the USA. Other sequences examined in this study also exhibited close connections to rosaviruses found in Hong Kong, China. Additionally, our sequences, while closely related to those of rosaviruses sampled from wild small mammals, displayed greater evolutionary divergence from those found in humans.

### Coevolutionary analysis

**Figure 4:**
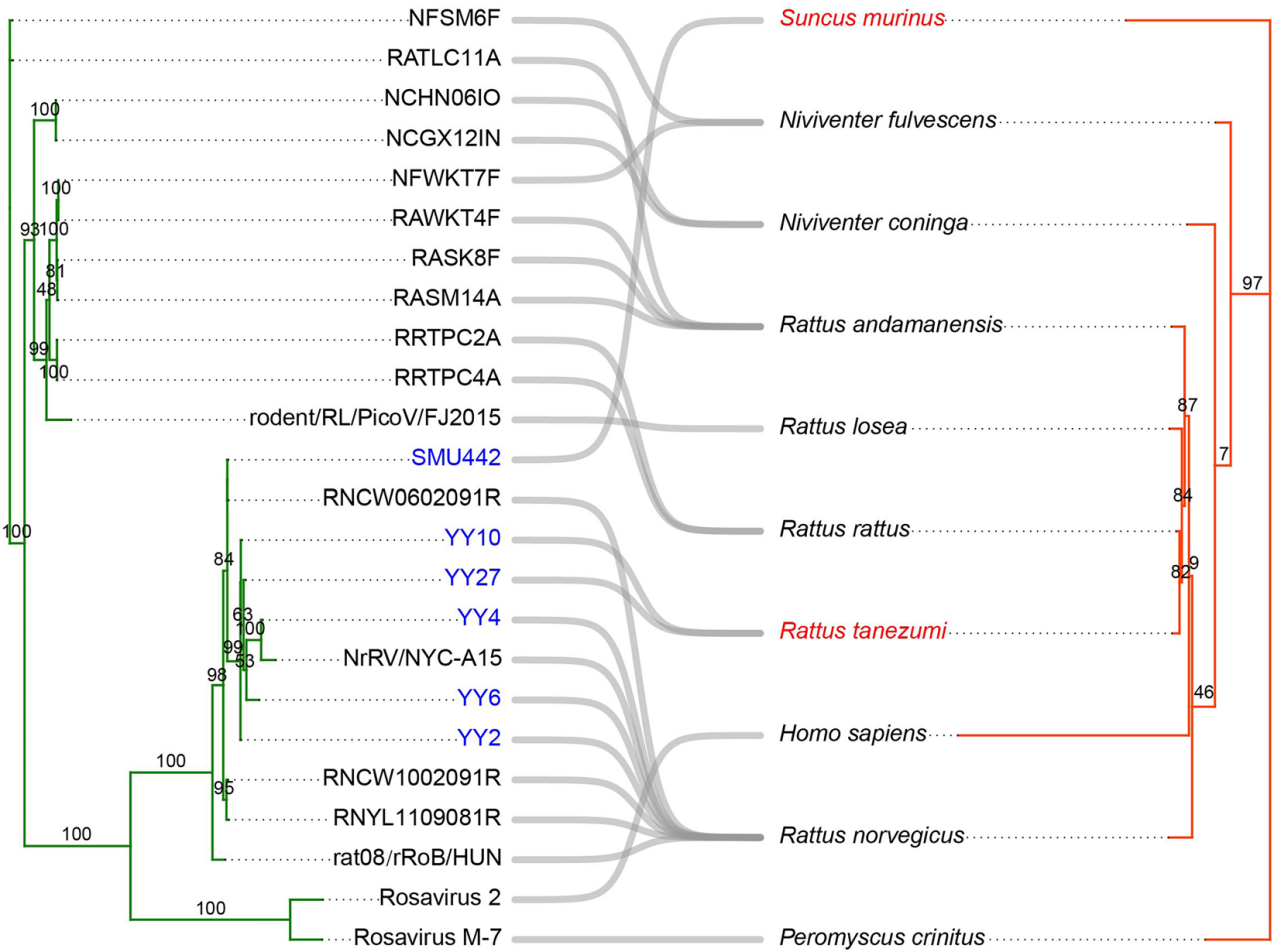
**Tanglegram of cophylogenetic relationships between rosaviruses (left) and their hosts (right)**. The tree was generated using the “cophylo” function of the *phytools* package in R. The numbers on the branches represent bootstrap values. The sequences and novel host species identified in this study are labelled with blue font and red font, respectively. The corresponding accession number in GenBank for each sequence is shown in Additional file 2.

**Figure 5:**
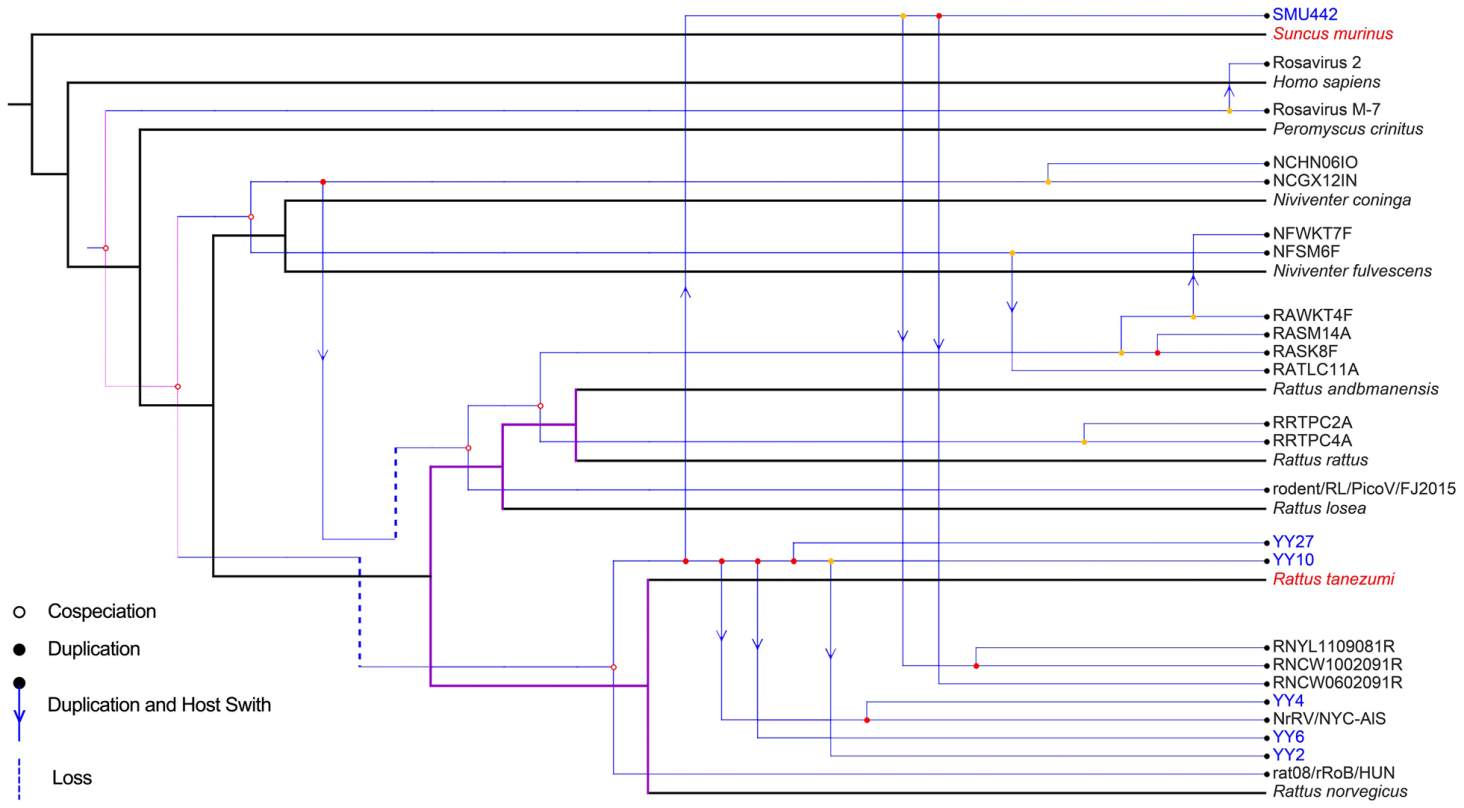
**Cophylogenetic analysis of rosaviruses and their hosts**. The blue branches represent the virus phylogeny, and the black branches represent the host phylogeny. Cospeciation events, duplication events, host switching events, and loss events are labelled with empty circles, filled circles, arrows, and dotted lines, respectively. The sequences and novel host species identified in this study are labelled with blue font and red font, respectively. The corresponding accession number in GenBank for each sequence is shown in Additional file 2.

The coevolutionary relationships between rosaviruses and their hosts are illustrated in Figure 4. The global fit test did not support substantial congruence between the phylogenies of rosaviruses and their hosts (*P* = 0.063), demonstrating that cospeciation is not the primary force driving pathogen diversity and host distribution. Using an event-based approach, we assembled the virus with its respective host at the order level. As depicted in Figure 5, the reconstruction identified six cospeciation events, ten host-switching events, three duplication events, and two loss events. Currently, rosaviruses have been found in three different orders of host species: Eulipotyphla, Primates, and Rodentia. Notably, SMU442 was discovered in *Suncus murinus*, a member of the order Eulipotyphla, thereby expanding the known range of hosts susceptible to rosaviruses. Similarly, this study represents the first detection of rosaviruses in tanezumi rats.

### Recombination and mutation analyses

**Figure 6:**
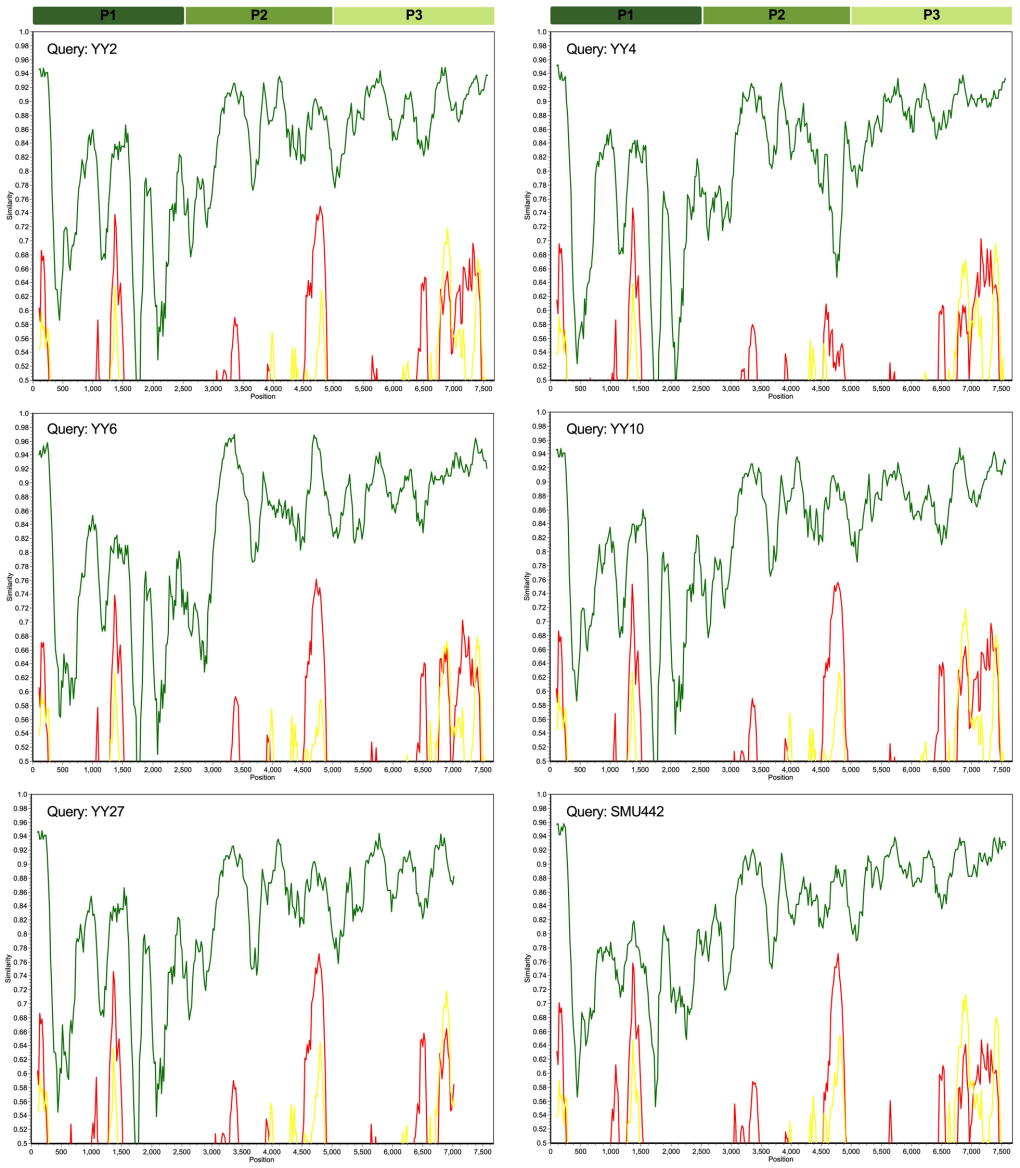
**Similarity plot of the complete polyprotein genomes of YY2, YY4, YY6, YY10, YY27, and SMU442 compared with those of other reference sequences**. Each point represents the similarity between the query sequence and a given heterologous sequence. The sequences identified in this study were used as the query sequences. Rosavirus A (accession no. KJ158169, yellow line), rosavirus B (MN116648, green line), and rosavirus C (KX783424, red line) were used as the reference sequences.

**Figure 7:**
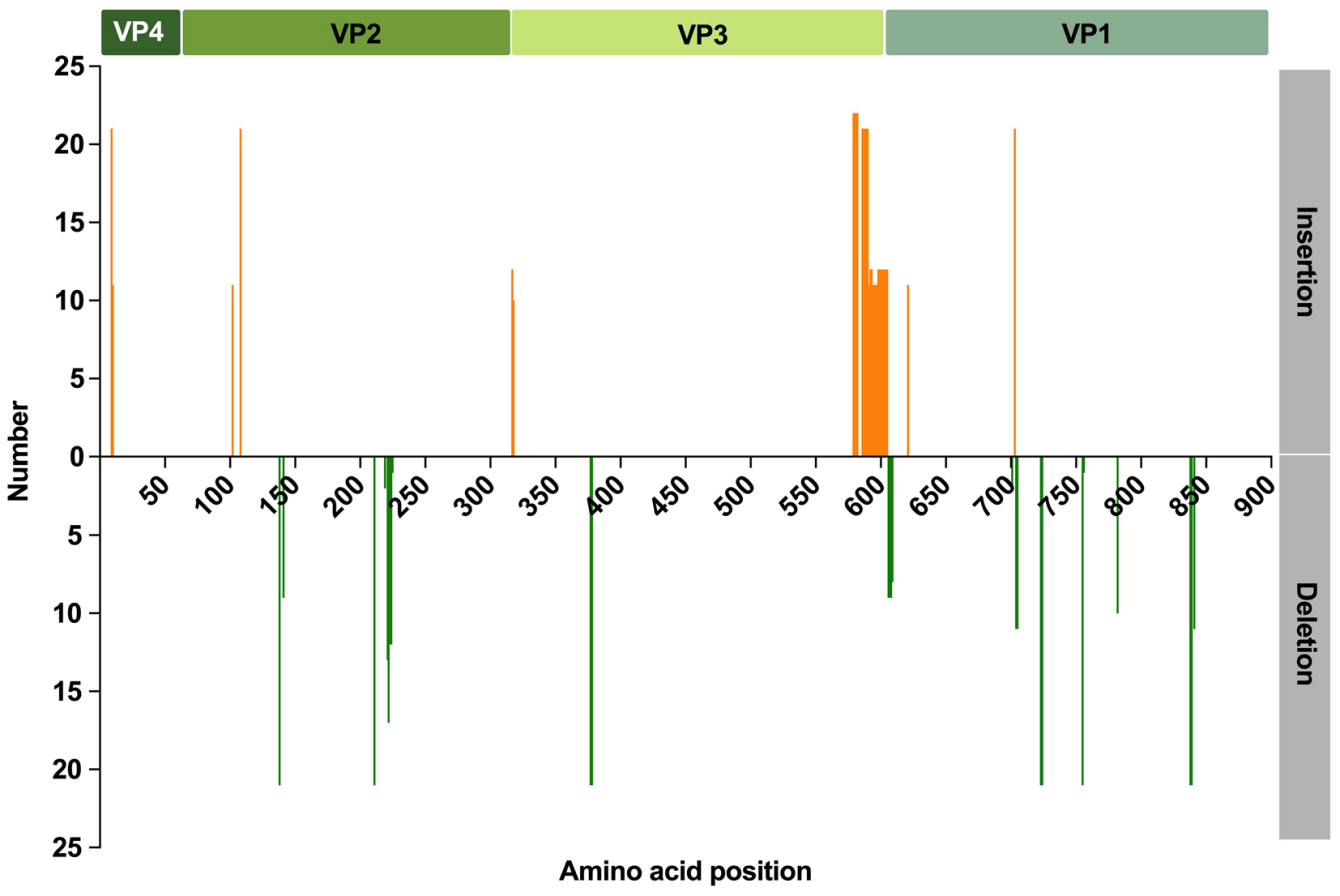
**Mutation scanning graphs of the rosavirus P1 gene**. The mutation sites were compared to those of the earliest strain, rosavirus M-7 (accession no. JF973686).

Recombination analysis based on full-length polyprotein genes revealed that no recombination events were detected for rosavirus. Nonetheless, the P1 coding region (895 aa) was referred to as having a greater range of genetic variability than the other reference sequences did (Figure 6). The P1 protein possibly mediates attachment of the rosavirus to cell surface receptors and fusion between the virus and cell membranes, similar to other picornaviruses. Consequently, we further scanned the mutation sites for the divergence of the P1 genes via BioAider. Overall, the VP1 subunit exhibited greater divergence than the other subunits did, accounting for the majority of the aa insertion and deletion mutants (Figure 7). Notably, the use of all available P1 sequences might lead to an overestimation of the number of synonymous and nonsynonymous mutations, even in cases where five major mutant types have been established. To address this, we restricted the analysis to P1 nt sequences of rosavirus B and compared them with the early virus strain RNYL1109081R (accession no. KX783422) in the GenBank database, with a total of 941 synonymous and nonsynonymous mutations being recognized (data not shown). Among these, 106 sites had both synonymous and nonsynonymous substitutions, with no termination mutation sites found. Notably, 193 nonsynonymous substitution sites resulted in alterations in the properties of the aa.

### Natural selection pressure

Both the M0 and two-ratio models were applied to determine whether individual branches were experiencing positive selection. The analytical findings revealed that positively selected pressures were more frequently found in rosavirus B and rosavirus C, as confirmed by the LRT (Table 3). A comparative analysis was performed to assess the selection pressures acting on rosavirus genotypes via the SLAC method. The results revealed that rosavirus B and rosavirus C are subjected primarily to negative selection, as indicated by the estimated *dN/dS* ratios (Table 4). Specifically, the *dN*/*dS* estimates were greater for the VP2-VP4, 2A, and 2C genes of rosavirus B than for those of rosavirus C. Conversely, the estimates of *dN*/*dS* values for the VP1, 2B, and 3A-3D segments of rosavirus B were lower than the elevated values observed for rosavirus C. Two sets of the M8 model and MEME approach were subsequently utilized to identify specific sites that were subjected to positive or relaxed selection. Our findings indicated that a greater proportion of sites in most genes of rosavirus B than in those of rosavirus C were likely under relaxed selection. However, the 2B, 3B, and 3D segments exhibited either an increase or no change in the sites under relaxed selection (Table 4).

**Table 3:** **Comparison of selection pressure in favour of branches for all genes of rosaviruses**

Gene	Lineages	Branch dN/dS by CODEML (two-ratio model)			M0 dN/dS	LRT *p* value
		Foreground clade	Background clade	Foreground clade/Background clade		
*VP4*	Rosavirus A	0.00244	0.02147	0.11365	0.01619	0.07298
	Rosavirus B	Inf	0.03237	–		0.08639
	Rosavirus C	0.20905	0.02124	9.84228		0.04742
*VP2*	Rosavirus A	0.0602	0.06555	0.91838	0.06554	0.94953
	Rosavirus B	0.01898	0.06603	0.28745		0.14109
	Rosavirus C	0.09494	0.06556	1.44814		0.76846
*VP3*	Rosavirus A	0.02321	0.03146	0.73776	0.03131	0.77632
	Rosavirus B	0.00199	0.03295	0.06039		0.00588
	Rosavirus C	0.00579	0.03232	0.17915		0.05785
*VP1*	Rosavirus A	0.0186	0.04235	0.43920	0.04214	0.66174
	Rosavirus B	0.00342	0.04278	0.07994		0.20414
	Rosavirus C	0.02652	0.04222	0.62814		0.75434
*2A*	Rosavirus A	0.00395	0.09937	0.03975	0.09837	0.06250
	Rosavirus B	0.00377	0.09857	0.03825		0.09902
	Rosavirus C	0.00361	0.10052	0.03591		< 0.001
*2B*	Rosavirus A	0.03478	0.06264	0.55524	0.06243	0.64805
	Rosavirus B	0.01216	0.06314	0.19259		0.17781
	Rosavirus C	0.00445	0.06269	0.07098		0.07818
*2C*	Rosavirus A	0.00698	0.03294	0.21190	0.03196	0.09941
	Rosavirus B	0.00132	0.03292	0.04010		0.01318
	Rosavirus C	0.00254	0.0326	0.07791		0.01976
*3A*	Rosavirus A	0.00837	0.06161	0.13585	0.05891	0.07310
	Rosavirus B	0.00595	0.05965	0.09975		0.30844
	Rosavirus C	0.00676	0.05985	0.11295		0.60332
*3B*	Rosavirus A	Inf	0.04608	–	0.04333	0.01093
	Rosavirus B	0.09585	0.03828	2.50392		0.27409
	Rosavirus C	0.0001	0.04368	0.00229		0.73119
*3C*	Rosavirus A	0.00272	0.04324	0.06290	0.04152	0.05516
	Rosavirus B	0.19566	0.04039	4.84427		0.34093
	Rosavirus C	0.07429	0.04082	1.81994		0.42575
*3D*	Rosavirus A	0.00139	0.03851	0.03609	0.03686	< 0.001
	Rosavirus B	0.0016	0.03801	0.04209		< 0.001
	Rosavirus C	0.00153	0.03732	0.04100		0.18465

Inf indicates zero synonymous changes, and the value equals infinity.

**Table 4:** **Comparison of selection pressure for all genes of rosaviruses**

Gene	Lineages	Global *dN/dS* by SLAC	Number of positively selected sites by M8 + BEB (amino-acid position)	Number of positively selected sites by MEME (amino-acid position)	Number of sites under relaxed selection
*VP4*	Rosavirus B	0.0906	1 (22)	0	1
	Rosavirus C	0	0	0	0
*VP2*	Rosavirus B	0.104	6 (69, 75, 77, 145, 147, 156)	2 (152, 212*)	6
	Rosavirus C	0.0907	2 (87*, 151)	3 (10, 40*, 101*)	2
*VP3*	Rosavirus B	0.0705	8 (74*, 178*, 239, 256, 257, 258, 259, 267*)	2 (259*, 267*)	6
	Rosavirus C	0.0599	0	3 (3*, 256*, 260*)	0
*VP1*	Rosavirus B	0.067	2 (81, 135)	3 (10*, 86, 246*)	2
	Rosavirus C	0.0751	0	4 (53*, 234*, 238, 247)	0
*2A*	Rosavirus B	0.141	9 (19, 42, 53, 65, 69, 74, 95, 133, 192)	3 (28*, 63*, 192*)	8
	Rosavirus C	0.127	3 (239, 250, 275)	8 (2*, 5*, 17*, 95*, 102*, 194*, 212*, 239*)	2
*2B*	Rosavirus B	0.0684	0	0	0
	Rosavirus C	0.13	9 (6, 15, 152, 169, 170*, 171, 172, 175, 177*)	3 (94*, 152*, 179)	8
*2C*	Rosavirus B	0.0614	3 (61, 277, 321)	2 (176, 204*)	3
	Rosavirus C	0.0437	0	4 (6, 203*, 270, 327*)	0
*3A*	Rosavirus B	0.0752	2 (27*, 62*)	1 (25*)	2
	Rosavirus C	0.0845	0	2 (84*, 87)	0
*3B*	Rosavirus B	0.0385	0	0	0
	Rosavirus C	0.121	0	0	0
*3C*	Rosavirus B	0.0273	1 (192)	1 (131*)	1
	Rosavirus C	0.0631	0	7 (37, 45, 60, 75*, 177, 192, 198*)	0
*3D*	Rosavirus B	0.0295	4 (41, 50, 55, 466*)	1 (50*)	3
	Rosavirus C	0.0679	4 (44, 78, 145, 370)	3 (134, 194, 430*)	4

Sites with asterisks (*) indicate statistical significance.

Moreover, the results of the MEME analysis suggested a predominance of positively selected sites in all genes of rosavirus C, apart from the VP4 gene. In this case, the 2A genes of rosavirus B and rosavirus C included a total of 3 and 8 positively selected sites, respectively, with considerable significance. The positive selection in rosavirus C appears to have been driven predominantly by the process of adapting to new host species.

### Time-scale evolutionary characterization of the rosavirus VP1 gene

**Figure 8:**
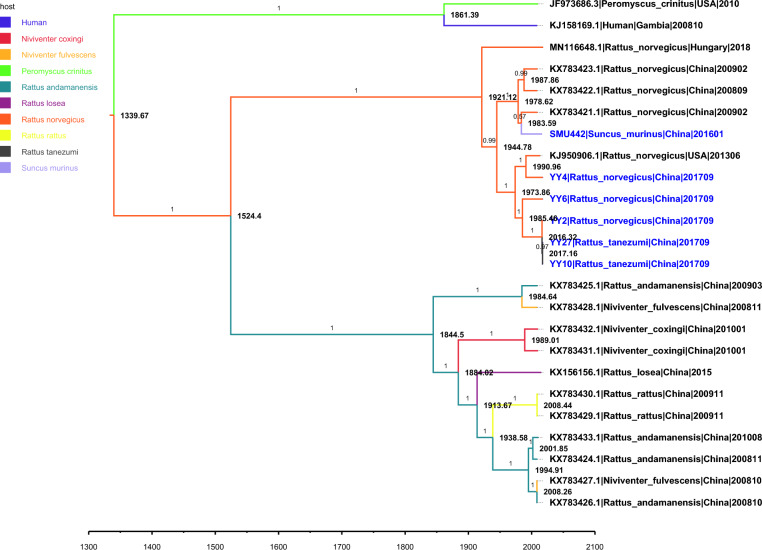
**Maximum clade credibility (MCC) tree representing the time-scale phylogeny of rosaviruses on the basis of the VP1 gene**. The times of divergence estimation are marked near the internal node. The numbers on branches represent posterior probabilities, and the colours of branches represent their host species, with the keys for colours shown on the left. The scale bars indicate the branch lengths by year. The accession numbers with the host species, isolation geographics, and collection dates are shown at the tips. The sequences identified in this study are labelled in blue.

A Bayesian MCMC approach was employed to construct a time-scale phylogenetic tree, with the aim of investigating the origin and evolutionary history of rosaviruses (Figure 8). The best-fitting model for the VP1 gene was GTR + G, which incorporates a strict clock and a coalescent Bayesian skyline. The tMRCA of the rosavirus VP1 gene was estimated to be in the year 1339.67 (95% highest posterior density [HPD], 874.91–1607.84). Compared with those of the other genotypes, the total number of rosavirus C sequences presented greater divergence, as evidenced by an estimated tMRCA of 1844.50 (95% HPDs, 1739.07–1910.94). Conversely, the time-scaled phylogeny of rosavirus A and rosavirus B predicted a tMRCA for each genotype in 1861.39 (95% HPDs, 1760.45 to 1923.95) and 1921.12 (95% HPDs, 1856.04 to 1957.99), respectively. The overall evolutionary rate of the VP1 gene was determined to be approximately 2.12 × 10^–3^ substitutions/site/year (95% HPDs, 1.09 × 10^–3^ to 3.00 × 10^–3^ substitutions/site/year). Additionally, the projected evolutionary rates of the three genotypes of rosavirus were similar.

### Phylogeographic inference and cross-species transmission of rosaviruses

**Figure 9:**
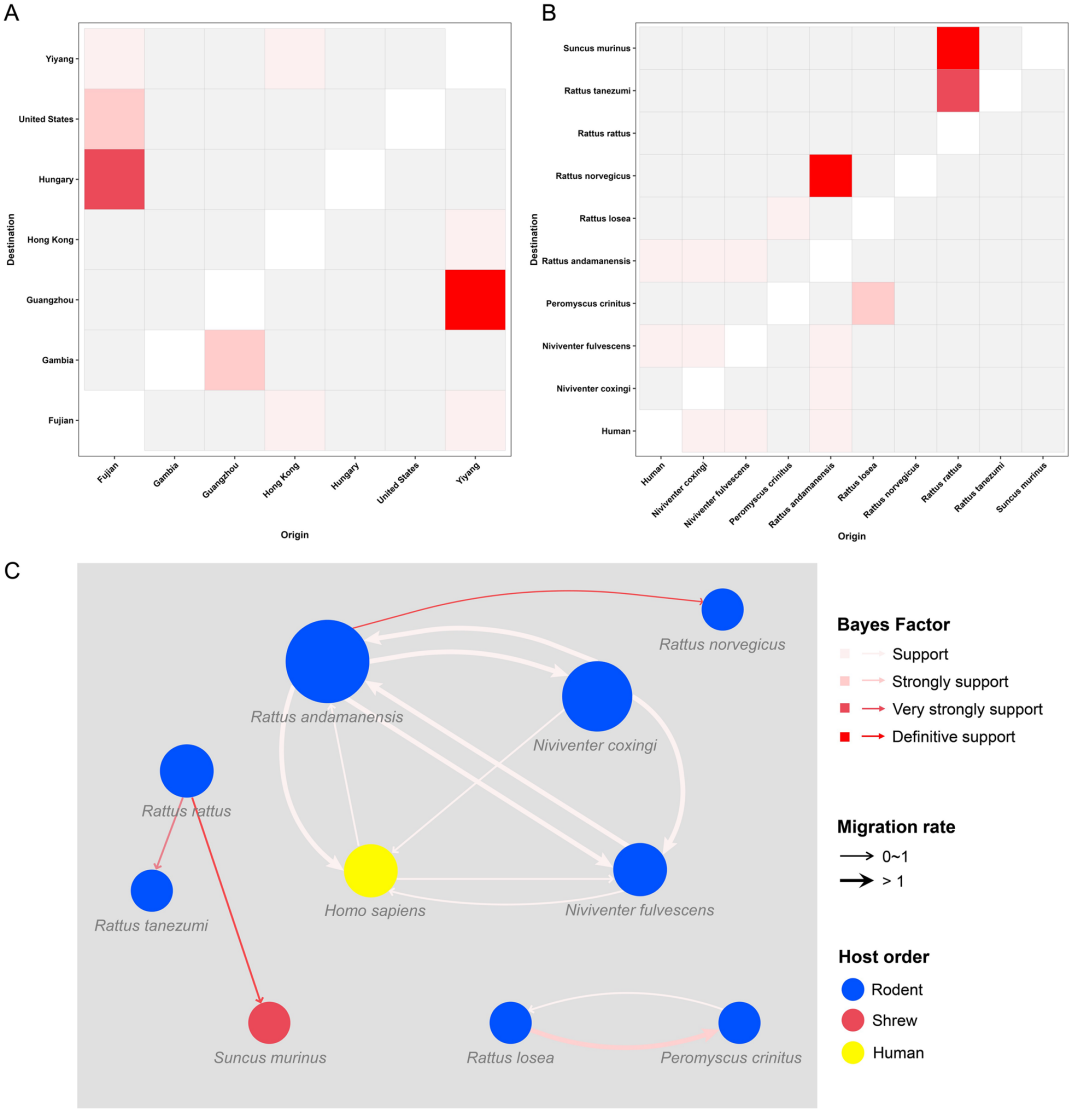
**Spatiotemporal diffusion of rosaviruses in different regions and hosts**. (**A**) Sufficient transmission routes inferred from the VP1 gene at different locations. (**B**) Sufficient transmission routes inferred from the VP1 gene in different hosts. (**C**) Transmission network of rosaviruses in different hosts. The different colours of the grid and arrows represent the Bayes factor values. The size of the circles represents the number of transmission routes spread from each species. Arrows indicate the direction of transmission between species, and arrows of different widths represent the migration rate.

The BSSVS procedure for MCMC time-scaled phylogenies was subsequently utilized to elucidate the worldwide spatial dissemination network and zoonotic origin of the genus *Rosavirus*. Overall, seven discrete sampling locations and five significant transmission pathways for geographic distribution were identified (Figure 9A; Additional file 5). This work provided robust evidence supporting migration routes from Fujian to Hungary (BF > 30) and to the USA (10 < BF < 30) in an east‒west direction. Similarly, a highly supported migration link was identified from Guangzhou to Gambia (10 < BF < 30). Definitively supported movement within China was observed from Yiyang to Guangzhou (BF > 100), with an additional support transition for the spread from Yiyang to Fujian (3 < BF < 10).

Given the dissemination networks of rosaviruses throughout different regions, regions inside China were more likely to export viruses to other sites than to import them. However, this may be subject to bias due to different migration rates and indicators, particularly resulting from disparities in sampling locations and varying sample sizes. Hence, it is highly important to ascertain additional rosavirus genome sequences to establish more robust migration connections.

With respect to the host species, ten discrete sampling host species and four notable transmission routes were identified (Figure 9B and C; Additional file 6). The Norway rat is considered the most likely ancestral host species of rosavirus, although its posterior probability is rather low at 0.231. Rosaviruses derived from tanezumi rats (YY10 and YY27) and Asian house shrews (SMU442) may be transmitted through roof rats, with posterior probabilities of 0.909 and 0.993, respectively. These findings support the occurrence of interspecies and cross-species transmission of rosaviruses from rodents. Rosavirus transmission from Indochinese forest rats to Norway rats was observed with definitive support (BF > 100). In addition, there was evidence of transmission from lesserricefield rats to canyon mice with a supported transition (3 < BF < 10). Notably, there is a frequent occurrence of host jumps of rosaviruses between rodents and humans (Figure 9C). These findings suggest an increased probability of cross-species transmission for rodent-derived rosaviruses and a potential concern regarding their spread to humans.

## Discussion

This study offers a comprehensive view of the epidemiology, genetic diversity, evolution, and potential transmission routes of rosaviruses. To the best of our knowledge, we present the first evidence of the presence of rosaviruses in tanezumi rats and Asian house shrews, illustrating that virus spillover or host switching between different host species may have occurred. Ongoing monitoring of animals living in both wildlife and close proximity to humans worldwide clearly remains highly important for understanding the evolutionary patterns of rosaviruses.

We identified a significant prevalence of rosaviruses among rodents and shrews in southern China, with an overall prevalence rate of 32.49%, as detailed in Table 1. This finding indicates substantial circulation of these viruses within rodent and shrew populations. The prevalence observed in this study was notably higher than that reported in previous research, where rosaviruses were detected in 5.71% and 5.07% of the potential alimentary and respiratory samples, respectively [9]. Specifically, our findings demonstrated a detection rate of 48.25% in Norway rats, 45.45% in tanezumi rats, and a markedly lower rate of 1.77% in Asian house shrews (Table 1). The absence of rosavirus detection in lesser ricefield rats and greater bandicoot rats suggested potential host-specific restrictions or differences in virus exposure among rodent species. This finding was restricted by the limited sample size. These data highlight the variations in the prevalence of rosaviruses among different host species and suggest that Norway rats and tanezumi rats may play a more critical role in the maintenance and transmission of rosaviruses. Ongoing epidemiological surveillance needs to be performed in an augmented effort due to the lack of data on infection rates of rosaviruses. Such efforts are essential to enhance our understanding of the epidemiology, host range, and transmission dynamics of rosaviruses, ultimately contributing to better prevention and control strategies for potential zoonotic threats.

To characterize genetic diversity and evolution further, we amplified six complete or nearly complete genome sequences of rosaviruses isolated from rodents and shrews in southern China. The *Picornaviridae* Study Group of ICTV officially determined that members of a particular species within the genus *Rosavirus* possess an identical genome structure, share more than 75% identity in the 2C and 3CD segments, and display less than 30% divergence in the P1 polyprotein aa sequences [39]. Accordingly, our sequences are classified within the same species as NrRV/NYC-A15 (accession no. KJ950906), RNCW1002091R (KX783421), and rat08/rRoB/HUN (MN116648). To ensure a more detailed categorization, Bayesian phylogenetic analyses were performed using 2C (Figure 2A) and 3CD (Figure 2B) aa sequences. The results revealed that our sequences formed a distinct cluster within the primary clade of rosaviruses, irrespective of their origin from different host species, and were clearly separated from other picornaviruses. On the basis of the phylogeny of P1 aa sequences, our sequences were determined to belong to the rosavirus B genotype (Figure 3). Genomic analysis revealed that our sequences were highly similar to those of rosavirus B and were significantly different from those of other members of the genus *Rosavirus*, with amino acid (aa) identities under 60% in all genes of P1, P2, P3, and the polyprotein (Figure 1).

The analysis of the rosavirus polyprotein sequences from our study revealed several key insights into the protease-cleavage sites, which are crucial for understanding the processing and maturation of viral proteins (Table 2). A unique cleavage site (E/S) identified in SMU442 between VP3 and VP1 may indicate a potential adaptation mechanism or evolutionary divergence. Notably, the cleavage sites between 3A and 3B (E/G), 3B and 3C (E/G), and 3C and 3D (G/L) are highly conserved across the rosavirus sequences, regardless of the host species. The conserved nature of these cleavage sites within the P3 region suggests a critical role in maintaining the functionality and stability of the viral replication machinery. These findings highlight the importance of proteolytic processing of the polyprotein into individual functional units [40]. Further studies are needed to elucidate the functional implications of these cleavage sites and their role in viral pathogenicity and host adaptation.

The detection of viral genomes exhibiting substantial homology in both rodents and shrews suggests the probability of viral evolution and spillover between different species. This argument is supported by cophylogenetic analyses, indicating that a host switch is a more appropriate interpretation for speciation within the genus *Rosavirus* (Figures 4, 5). Additionally, the cophylogenetic tree revealed that closely clustered rosaviruses, such as coxing white-bellied rats and chestnut white-bellied rats, which belong to the same host genus as *Niviventer,* are generally found in closely related rodent species (Figure 4). More precisely, these rodent species contain rosavirus sequences such as NFSM6F (accession no. KX783428), NFWKT7F (KX783427), RATLC11A (KX783425), NCHN06IO (KX783431), and NCGX12IN (KX783432). Comprehending the coevolutionary relationship between rosaviruses and their hosts provides valuable insights into viral transmission between wild small mammals and humans and serves as a reference for monitoring efforts related to their spread [41].

Despite the absence of clear evidence for homologous recombination among rosaviruses in this study, P1 proteins, which have relatively high genetic variability, contain the most diverse motif responsible for encoding the viral capsid protein (Figure 6). This motif likely plays a crucial role in determining the biology, pathogenicity, and antigenicity of non-enveloped viruses, similar to observations in other picornaviruses [42–44]. Viral capsids, which are the primary focus of the host’s immune system, undergo a constant process of modification to evade detection by introducing mutations into the genes encoding the viral capsid proteins [45]. However, the comprehensive structure and functions of capsids in rosaviruses remain inadequately understood. In this study, we analysed twenty-three P1 genomes of rosaviruses and detected several insertion and deletion mutants, primarily in the VP1 gene (Figure 7). Rodents and shrews act as the primary reservoir for diverse picornaviruses that can cross species boundaries through recombination or mutation. The accumulation of mutations in the P1 protein is likely linked to alterations in host specificity, as this gene plays a crucial role in the entry of picornaviruses. These traits may facilitate the transmission of rosaviruses from animals to humans, as evidenced by previous observations of animal coronaviruses [46]. As a result, it is imperative to closely monitor the genetic variations in these viruses and conduct thorough screenings for sites with functional mutations to effectively prevent and control the spread of emerging infectious diseases.

The heterogeneities in both the nt and aa sequences were significant across rosaviruses, with rosavirus C species exhibiting greater diversity than other genotypes (Table 3). These findings suggest that rosavirus C has undergone a distinct evolutionary process. A comprehensive analysis of selection pressure was executed for each viral gene by measuring the *dN/dS* ratio. Positive selection accelerates adaptive mutations that increase viral survival and transmission [47]. Notably, we observed more elevated *dN/dS* estimates in rosavirus B, which may be a consequence of relaxed selective constraints following cross-species transmission, which is consistent with the wide range of host species for this genotype (Table 4). The conserved domain (3D), which is responsible for encoding RNA-dependent RNA polymerase (RdRp), underwent an increase in positive selection pressure, potentially increasing the replication capacity of viral genomes [48]. However, further experimental research is necessary to elucidate the precise biological function and mechanism of these sites.

In our study, along with earlier ones, we identified rosaviruses that can be categorized into three primary genotypes, which are found in humans, Asian house shrews, and various rodent species. We determined that the VP1 sequences of rosaviruses originated around the year 1339 (Figure 8). The overall evolutionary rate was calculated to be 2.12 × 10^–3^ substitutions/site/year, similar to the evolutionary rates recognized in related picornaviruses [36]. Furthermore, the origin of the rosavirus B clade approximately 1921 reflects its relative youth compared with other clades (Figure 8). Notably, the analysed dataset may have temporal bias due to its small size. To mitigate this concern, we considered the previous distribution of the evolutionary rate of relevant picornaviruses. Nevertheless, these estimations may still be influenced by the number of sequences and the specific genomic region analysed. Future studies should address these aspects to provide more accurate and comprehensive evolutionary insights.

Our study clarifies the movement of rosaviruses throughout different regions and elucidates the possible transmission network among diverse host species. These findings reveal that rosaviruses tend to disseminate globally and facilitate a broad east‒west distribution, including pathways from Fujian to Hungary and to the USA (Figure 9A; Additional file 5). Additionally, the presence of two prominent migration links from Yiyang to Guangzhou and to Fujian highlights a substantially greater degree of gene flow within China (Figure 9A; Additional file 5). These data support the notion that rosaviruses can transmit over both short and long distances. Zeller et al. reported that international travel driven by commerce and economic movement has resulted in the dispersal of disease-causing agents across significant distances between different locations [49]. However, notably, a substantial proportion of viral sequences have been identified in China. This may not accurately depict the true global situation because of inadequate molecular investigations in many parts of the world. The sample imbalance creates ambiguities regarding the spatial dispersion of the virus at the global level. Hence, obtaining a more comprehensive dataset of rosaviruses from various geographic regions and host species is essential to strengthening the foundation for understanding virus transmission dynamics.

In terms of the host origin of rosaviruses, although they are believed to have originated from Norway rats with a lower posterior probability, there is substantial evidence of cross-species transmission events (Figure 9B and C; Additional file 6). Among them, there was a notable host jump from the Indochinese forest rat to the Norway rat (BF > 3 and posterior probability > 0.5). The newly discovered hosts of rosaviruses, tanezumi rats and Asian house shrews, are most likely to have been infected by roof rats (BF > 30). This observation aligns with the fact that viruses in rodents are remarkably more likely to be transmitted to other host species than are viruses in other wild small mammals, such as bats and shrews [50]. These data substantiated the occurrence of interspecies and cross-species transmission from rodents (Additional file 6).

Notably, several small mammals, specifically the Norway rat, tanezumi rat, and Asian house shrew, are peridomestic or synanthropic species that commonly reside alongside humans throughout Southeast Asia. Some of these species are familiar with medically relevant pathogens that can be transmitted to humans via urine or faeces [51]. Asian house shrews, in particular, have been implicated as hosts for various viruses, including coronaviruses, hantaviruses, and hepatitis E virus, and serve as important reservoirs for zoonotic infections [52]. Our findings suggest that Asian house shrews contributed significantly to the evolution and cross-species transmission of rosaviruses. Conducting ongoing surveillance research on shrews in various settings will enable a richer understanding of the precise role that Asian house shrews play in the evolution of rosaviruses. Another notion is that potential transmission links (BF > 3) could connect human populations from Indochinese forest rats, coxing white-bellied rats, and chestnut white-bellied rats. This finding aligns with previous research indicating that a rosavirus strain identified in a child with diarrhoea may arise from a rodent species [8]. These data underscore the zoonotic potential of rosaviruses and their ability to cause human infections through spillover events [53, 54].

Our findings provide more evidence that rosaviruses are transmitted between different hosts and subsequently adapt to novel mammalian orders, suggesting that these viruses can overcome host genetic barriers. As such, this study underscores the necessity of broadening virus monitoring efforts to include a wider range of geographic regions and host species, which is crucial for comprehensively understanding the transmission patterns and evolutionary trajectories of rosaviruses.

In conclusion, this molecular epidemiology study reveals the extensive presence of rosaviruses in rats and shrews from southern China, providing valuable insight into the occurrence of viral dispersal. The identification of rosavirus B in tanezumi rats and Asian house shrews enhances our understanding of its evolutionary history, indicating a wider host spectrum than previously known. Further genomic and evolutionary research is essential to ascertain their geographical spread, genetic diversity, and long-term population-level dynamics.

## Supplementary Information

Additional file 1. Information for primer sequences.

Additional file 2. Sequences used in the present study.

Additional file 3. Marginal likelihoods estimated via molecular clock models and coalescent models.

Additional file 4. Mean pairwise amino acid identity of the P1 and P3 proteins of rosaviruses. **P < 0.01, ***P < 0.001, ****P < 0.0001.

Additional file 5. Migration paths of rosaviruses in seven geographical locations.

Additional file 6. Cross-species transmission of rosaviruses in 10 host species

## Data Availability

The complete or nearly complete genomes of the rosavirus sequences in this study have been deposited in the GenBank database under accession numbers PQ045667 to PQ045672. The full datasets used and analysed during the current study are available from the corresponding author upon reasonable request.

## Abbreviations

SARS-CoV: severe acute respiratory syndrome coronavirus
MERS-CoV: Middle East respiratory coronavirus
SARS-CoV-2: severe acute respiratory syndrome coronavirus 2
ICTV: International Committee on Taxonomy of Viruses
USA: United States of America
Cytb: mitochondrial cytochrome b
PBS: phosphate-buffered saline
nt: nucleotide
aa: amino acid
ORF: open reading frame
BLAST: basic local alignment search tool
NCBI: National Center for Biotechnology Information
RDP: recombination detection program
SLAC: single-likelihood ancestor counting
*dN/dS*: nonsynonymous to synonymous
LRT: likelihood ratio test
MEME: mixed effects model of evolution
MCMC: Bayesian Monte Carlo Markov chain
tMRCA: time to the most recent common ancestor
HPD: highest posterior density
ESS: effective sample size
MCC: maximum clade credibility
BSSVS: Bayesian stochastic search variable selection
BF: Bayes factor
RdRp: RNA-dependent RNA polymerase

## References

[1] Memish ZA, Mishra N, Olival KJ, Fagbo SF, Kapoor V, Epstein JH, Alhakeem R, Durosinloun A, Al Asmari M, Islam A, Kapoor A, Briese T, Daszak P, Al Rabeeah AA, Lipkin WI (2013) Middle East respiratory syndrome coronavirus in bats, Saudi Arabia. Emerg Infect Dis 19:1819–1823 24206838 10.3201/eid1911.131172 PMC3837665

[2] Lu R, Zhao X, Li J, Niu P, Yang B, Wu H, Wang W, Song H, Huang B, Zhu N, Bi Y, Ma X, Zhan F, Wang L, Hu T, Zhou H, Hu Z, Zhou W, Zhao L, Chen J, Meng Y, Wang J, Lin Y, Yuan J, Xie Z, Ma J, Liu WJ, Wang D, Xu W, Holmes EC, Gao GF, Wu G, Chen W, Shi W, Tan W (2020) Genomic characterisation and epidemiology of 2019 novel coronavirus: implications for virus origins and receptor binding. Lancet 395:565–574 32007145 10.1016/S0140-6736(20)30251-8 PMC7159086

[3] Zhuo X, Feschotte C (2015) Cross-species transmission and differential fate of an endogenous retrovirus in three mammal lineages. PLoS Pathog 11:e1005279 26562410 10.1371/journal.ppat.1005279 PMC4643047

[4] Taylor LH, Latham SM, Woolhouse ME (2001) Risk factors for human disease emergence. Philos Trans R Soc Lond B Biol Sci 356:983–989 11516376 10.1098/rstb.2001.0888 PMC1088493

[5] Picornaviruses Home. https://www.picornaviridae.com/. Accessed 12 July 2023

[6] Phan TG, Kapusinszky B, Wang C, Rose RK, Lipton HL, Delwart EL (2011) The fecal viral flora of wild rodents. PLoS Pathog 7:e1002218 21909269 10.1371/journal.ppat.1002218 PMC3164639

[7] Phan TG, Vo NP, Simmonds P, Samayoa E, Naccache S, Chiu CY, Delwart E (2013) Rosavirus: the prototype of a proposed new genus of the *Picornaviridae* family. Virus Genes 47:556–558 23943414 10.1007/s11262-013-0968-1 PMC4160104

[8] Lim ES, Cao S, Holtz LR, Antonio M, Stine OC, Wang D (2014) Discovery of rosavirus 2, a novel variant of a rodent-associated picornavirus, in children from The Gambia. Virology 454–455:25–33 24725928 10.1016/j.virol.2014.01.018 PMC4096378

[9] Lau SK, Woo PC, Li KS, Zhang HJ, Fan RY, Zhang AJ, Chan BC, Lam CS, Yip CC, Yuen MC, Chan KH, Chen ZW, Yuen KY (2016) Identification of novel rosavirus species that infects diverse rodent species and causes multisystemic dissemination in mouse model. PLoS Pathog 12:e1005911 27737017 10.1371/journal.ppat.1005911 PMC5063349

[10] Boros Á, Orlovácz K, Pankovics P, Szekeres S, Földvári G, Fahsbender E, Delwart E, Reuter G (2019) Diverse picornaviruses are prevalent among free-living and laboratory rats (*Rattus norvegicus*) in Hungary and can cause disseminated infections. Infect Genet Evol 75:103988 31377399 10.1016/j.meegid.2019.103988

[11] Okitsu S, Khamrin P, Takanashi S, Thongprachum A, Hoque SA, Takeuchi H, Khan MA, Hasan SMT, Iwata T, Shimizu H, Jimba M, Hayakawa S, Maneekarn N, Ushijima H (2020) Molecular detection of enteric viruses in the stool samples of children without diarrhea in Bangladesh. Infect Genet Evol 77:104055 31629889 10.1016/j.meegid.2019.104055

[12] Thongprachum A, Fujimoto T, Takanashi S, Saito H, Okitsu S, Shimizu H, Khamrin P, Maneekarn N, Hayakawa S, Ushijima H (2018) Detection of nineteen enteric viruses in raw sewage in Japan. Infect Genet Evol 63:17–23 29753903 10.1016/j.meegid.2018.05.006

[13] Wang W, Lin XD, Zhang HL, Wang MR, Guan XQ, Holmes EC, Zhang YZ (2020) Extensive genetic diversity and host range of rodent-borne coronaviruses. Virus Evol 6:veaa078 33318860 10.1093/ve/veaa078 PMC7665783

[14] Benchling website. http://benchiling.com. Accessed 15 July 2023

[15] National Center for Biotechnology Information. https://www.ncbi.nlm.nih.gov/. Accessed 16 July 2023

[16] Katoh K, Standley DM (2013) MAFFT multiple sequence alignment software version 7: improvements in performance and usability. Mol Biol Evol 30:772–780 23329690 10.1093/molbev/mst010 PMC3603318

[17] Open Reading Frame Finder. https://www.ncbi.nlm.nih.gov/orffinder. Accessed 17 July 2023

[18] Zhou ZJ, Qiu Y, Pu Y, Huang X, Ge XY (2020) BioAider: An efficient tool for viral genome analysis and its application in tracing SARS-CoV-2 transmission. Sustain Cities Soc 63:102466 32904401 10.1016/j.scs.2020.102466 PMC7455202

[19] Ronquist F, Teslenko M, van der Mark P, Ayres DL, Darling A, Höhna S, Larget B, Liu L, Suchard MA, Huelsenbeck JP (2012) MrBayes 3.2: efficient Bayesian phylogenetic inference and model choice across a large model space. Syst Biol 61:539–542 22357727 10.1093/sysbio/sys029 PMC3329765

[20] Kalyaanamoorthy S, Minh BQ, Wong TKF, von Haeseler A, Jermiin LS (2017) ModelFinder: fast model selection for accurate phylogenetic estimates. Nat Methods 14:587–589 28481363 10.1038/nmeth.4285 PMC5453245

[21] Guangchuang Yu, Smith DK, Zhu H, Guan Yi, Lam TT-Y (2017) ggtree: an R package for visualization and annotation of phylogenetic trees with their covariates and other associated data. Methods Ecol Evol 8:28–36

[22] Shuangbin X, Li L, Luo X, Chen M, Tang W, Zhan L, Dai Z, Lam TT, Yi Guan YG (2022) Ggtree: a serialized data object for visualization of a phylogenetic tree and annotation data. iMeta 1:e56 38867905 10.1002/imt2.56 PMC10989815

[23] Wang LG, Lam TT, Xu S, Dai Z, Zhou L, Feng T, Guo P, Dunn CW, Jones BR, Bradley T, Zhu H, Guan Y, Jiang Y, Yu G (2020) Treeio: an R package for phylogenetic tree input and output with richly annotated and associated data. Mol Biol Evol 37:599–603 31633786 10.1093/molbev/msz240 PMC6993851

[24] Legendre P, Desdevises Y, Bazin E (2002) A statistical test for host-parasite coevolution. Syst Biol 51:217–234 12028729 10.1080/10635150252899734

[25] Conow C, Fielder D, Ovadia Y, Libeskind-Hadas R (2010) Jane: a new tool for the cophylogeny reconstruction problem. Algorithms Mol Biol 5:16 20181081 10.1186/1748-7188-5-16 PMC2830923

[26] Revell L (2012) Phytools: an R package for phylogenetic comparative biology (and other things). PeerJ 12:e16505 10.7717/peerj.16505 PMC10773453 38192598

[27] Martin DP, Lemey P, Lott M, Moulton V, Posada D, Lefeuvre P (2010) RDP3: a flexible and fast computer program for analyzing recombination. Bioinformatics 26:2462–2463 20798170 10.1093/bioinformatics/btq467 PMC2944210

[28] Kosakovsky Pond SL, Frost SD (2005) Not so different after all: a comparison of methods for detecting amino acid sites under selection. Mol Biol Evol 22:1208–1222 15703242 10.1093/molbev/msi105

[29] Delport W, Poon AF, Frost SD, Kosakovsky Pond SL (2010) Datamonkey 2010: a suite of phylogenetic analysis tools for evolutionary biology. Bioinformatics 26:2455–2457 20671151 10.1093/bioinformatics/btq429 PMC2944195

[30] Datamonkey Webserver. https://www.datamonkey.org/. Accessed 21 July 2023

[31] Yang Z (2007) PAML 4: phylogenetic analysis by maximum likelihood. Mol Biol Evol 24:1586–1591 17483113 10.1093/molbev/msm088

[32] Yang Z, Wong WS, Nielsen R (2005) Bayes empirical bayes inference of amino acid sites under positive selection. Mol Biol Evol 22:1107–1118 15689528 10.1093/molbev/msi097

[33] Drummond AJ, Suchard MA, Xie D, Rambaut A (2012) Bayesian phylogenetics with BEAUti and the BEAST 1.7. Mol Biol Evol 29:1969–1973 22367748 10.1093/molbev/mss075 PMC3408070

[34] Baele G, Lemey P, Bedford T, Rambaut A, Suchard MA, Alekseyenko AV (2012) Improving the accuracy of demographic and molecular clock model comparison while accommodating phylogenetic uncertainty. Mol Biol Evol 29:2157–2167 22403239 10.1093/molbev/mss084 PMC3424409

[35] Drummond AJ, Rambaut A, Shapiro B, Pybus OG (2005) Bayesian coalescent inference of past population dynamics from molecular sequences. Mol Biol Evol 22:1185–1192 15703244 10.1093/molbev/msi103

[36] Lu L, Van Dung N, Bryant JE, Carrique-Mas J, Van Cuong N, Anh PH, Rabaa MA, Baker S, Simmonds P, Woolhouse ME (2016) Evolution and phylogeographic dissemination of endemic porcine picornaviruses in Vietnam. Virus Evol 2:vew001 27774295 10.1093/ve/vew001 PMC4989877

[37] Lu L, Van Dung N, Ivens A, Bogaardt C, O’Toole A, Bryant JE, Carrique-Mas J, Van Cuong N, Anh PH, Rabaa MA, Tue NT, Thwaites GE, Baker S, Simmonds P, Woolhouse ME (2018) Genetic diversity and cross-species transmission of kobuviruses in Vietnam. Virus Evol 4:vey002 29449965 10.1093/ve/vey002 PMC5810437

[38] Rambaut A, Drummond AJ, Xie D, Baele G, Suchard MA (2018) Posterior summarization in bayesian phylogenetics using Tracer 1.7. Syst Biol 67:901–904 29718447 10.1093/sysbio/syy032 PMC6101584

[39] Zell R, Delwart E, Gorbalenya AE, Hovi T, King AMQ, Knowles NJ, Lindberg AM, Pallansch MA, Palmenberg AC, Reuter G, Simmonds P, Skern T, Stanway G, Yamashita T, Ictv Report C (2017) ICTV virus taxonomy profile: *Picornaviridae*. J Gen Virol 98:2421–2422 28884666 10.1099/jgv.0.000911 PMC5725991

[40] Petersen JF, Cherney MM, Liebig HD, Skern T, Kuechler E, James MN (1999) The structure of the 2A proteinase from a common cold virus: a proteinase responsible for the shut-off of host-cell protein synthesis. Embo J 18:5463–5475 10523291 10.1093/emboj/18.20.5463 PMC1171615

[41] Liang J, Zhu C, Zhang L (2021) Cospeciation of coronavirus and paramyxovirus with their bat hosts in the same geographical areas. BMC Ecol Evol 21:148 34325659 10.1186/s12862-021-01878-7 PMC8319908

[42] Rossmann MG, Arnold E, Erickson JW, Frankenberger EA, Griffith JP, Hecht HJ, Johnson JE, Kamer G, Luo M, Mosser AG, Rueckert R, Sherry B, Vriend G (1985) Structure of a human common cold virus and functional relationship to other picornaviruses. Nature 317:145–153 2993920 10.1038/317145a0

[43] Zhang M, Li Q, Wu F, Ou Z, Li Y, You F, Chen Q (2021) Epidemiology, Genetic Characterization, and evolution of *Hunnivirus* carried by *Rattus norvegicus* and *Rattus tanezumi:* the first epidemiological evidence from southern China. Pathogens 10:661 34071186 10.3390/pathogens10060661 PMC8226955

[44] Mattenberger F, Latorre V, Tirosh O, Stern A, Geller R (2021) Globally defining the effects of mutations in a picornavirus capsid. eLife 10:e64256 33432927 10.7554/eLife.64256 PMC7861617

[45] Cifuente JO, Moratorio G (2019) Evolutionary and structural overview of human picornavirus capsid antibody evasion. Front Cell Infect Microbiol 9:283 31482072 10.3389/fcimb.2019.00283 PMC6710328

[46] Gussow AB, Auslander N, Faure G, Wolf YI, Zhang F, Koonin EV (2020) Genomic determinants of pathogenicity in SARS-CoV-2 and other human coronaviruses. Proc Natl Acad Sci USA 117:15193–15199 32522874 10.1073/pnas.2008176117 PMC7334499

[47] Li G, Wang R, Cai Y, Zhang J, Zhao W, Gao Q, Franzo G, Su S (2020) Epidemiology and evolutionary analysis of Torque teno sus virus. Vet Microbiol 244:108668 32402339 10.1016/j.vetmic.2020.108668

[48] Lescar J, Canard B (2009) RNA-dependent RNA polymerases from flaviviruses and *Picornaviridae*. Curr Opin Struct Biol 19:759–767 19910184 10.1016/j.sbi.2009.10.011

[49] Zeller M, Heylen E, Damanka S, Pietsch C, Donato C, Tamura T, Kulkarni R, Arora R, Cunliffe N, Maunula L, Potgieter C, Tamim S, Coster SD, Zhirakovskaya E, Bdour S, O’Shea H, Kirkwood CD, Seheri M, Nyaga MM, Mphahlele J, Chitambar SD, Dagan R, Armah G, Tikunova N, Van Ranst M, Matthijnssens J (2015) Emerging OP354-Like P[8] rotaviruses have rapidly dispersed from Asia to other continents. Mol Biol Evol 32:2060–2071 25858434 10.1093/molbev/msv088 PMC4833074

[50] Chen YM, Hu SJ, Lin XD, Tian JH, Lv JX, Wang MR, Luo XQ, Pei YY, Hu RX, Song ZG, Holmes EC, Zhang YZ (2023) Host traits shape virome composition and virus transmission in wild small mammals. Cell 186:4662-4675.e12 37734372 10.1016/j.cell.2023.08.029

[51] Francis C (2019) Field guide to the mammals of South-east Asia. Bloomsbury Publishing, London

[52] Guo H, Cai C, Wang B, Zhuo F, Jiang R, Wang N, Li B, Zhang W, Zhu Y, Fan Y, Chen W, Chen W, Yang X, Shi Z (2019) Novel hepacivirus in Asian house shrew, China. Sci China Life Sci 62:701–704 30701456 10.1007/s11427-018-9435-7 PMC7088713

[53] Olival KJ, Hosseini PR, Zambrana-Torrelio C, Ross N, Bogich TL, Daszak P (2017) Host and viral traits predict zoonotic spillover from mammals. Nature 546:646–650 28636590 10.1038/nature22975 PMC5570460

[54] Plowright RK, Parrish CR, McCallum H, Hudson PJ, Ko AI, Graham AL, Lloyd-Smith JO (2017) Pathways to zoonotic spillover. Nat Rev Microbiol 15:502–510 28555073 10.1038/nrmicro.2017.45 PMC5791534

