# Supplementary material for: A panoramic view of the molecular epidemiology, evolution, and cross-species transmission of rosaviruses
Source: Vet Res. 2024 Nov 8;55:145. doi: 10.1186/s13567-024-01399-3 (PMC11545274; doi:10.1186/s13567-024-01399-3)
Supplement: Supplementary file 1 — Additional file 1. Information for primer sequences. [file 13567_2024_1399_MOESM1_ESM.docx]

**Additional file 1 Information for primer sequences**

| Reaction Number | Primer Name | Primer Sequences (5'-3') | Size (bp) |
| --- | --- | --- | --- |
| 1 | RoV-114-F | GTCTCTTTAGTGTCTATGCTTC | 475 |
|  | RoV-568-R | TTGCTGCCATTTCTGCCAGCCA |  |
| 2 | RoV-148-F | GACAGCGTTGGGCATCATGGCT | 534 |
|  | RoV-660-R | CTGCAATYTGRCCAACAACAGG |  |
| 3 | RoV-447-F | CCGTCTGTCCGGCGTAGTCAGA | 763 |
|  | RoV-1402-R | ACGGTRACKGGGGTRTTGGWGG |  |
| 4 | RoV-1330-F | TGGCTCACCACCACCGGTACAT | 604 |
|  | RoV-1909-R | CCAGCCATGGCCTGCTCCATYG |  |
| 5 | RoV-1843-F | CGACYGCGATGACTCGYGGYCG | 712 |
|  | RoV-2533-R | TAAAGCCGSCGYCTGCCAAGRA |  |
| 6 | rRoB-VP1-Fgen | ATCCACATGGGTGACTCAGAC | 623 |
|  | rRoB-VP1-Rgen | TARACGTTWGCAACGTTACTCCA |  |
| 7 | RoV-2533-F | TYCTTGGCAGRCGSCGGCTTTA | 934 |
|  | RoV-3445-R | WASCGRTCATCCCACTCGAGCT |  |
| 8 | RoV-3322-F | GTGTTGAAACCAGAWCCCGCCC | 736 |
|  | RoV-4036-R | GGAACCCAATTACGCGGCTCCC |  |
| 9 | RoV-3988-F | CTGTYCGTCTTGCAGCCTTTGG | 700 |
|  | RoV-4666-R | ATCCACTCGACRTTYCTTGCAG |  |
| 10 | RoV-4547-F | CCCCACCCTCCCCCTTTTGGAT | 908 |
|  | RoV-5433-R | AGGGACCAGTKGGGGTWAGGGC |  |
| 11 | RoV-4968-F | CGCGCTCGACAGGCTCAGTATG | 681 |
|  | RoV-5627-R | CCACTCACGCACWATKGGGTTY |  |
| 12 | RoV-5433-F | GCCCTAACCCCAACWGGWCCCT | 792 |
|  | RoV-6203-R | RCGCTCCACTCTCCKCATCTTC |  |
| 13 | RoV-6119-F | YCGCACGTACGTYTGCAATGCY | 839 |
|  | RoV-6936-R | CAGCTATGTAGGGYACACCGGG |  |
| 14 | RoV-6669-F | TCCCCMGCCTAYGGAGCYTTCC | 840 |
|  | RoV-7487-R | GAACACRCTAGTCCCGCAGCAG |  |
| 15 | RoV-7331-F | ATCYGTGCCAACYTGCGCCTTY | 705 |
|  | RoV-8014-R | YACRCCGGCACCAYAACCTCGA |  |
