# Supplementary material for: A panoramic view of the molecular epidemiology, evolution, and cross-species transmission of rosaviruses
Source: Vet Res. 2024 Nov 8;55:145. doi: 10.1186/s13567-024-01399-3 (PMC11545274; doi:10.1186/s13567-024-01399-3)
Supplement: Supplementary file 2 — Additional file 2. Sequences used in the present study. [file 13567_2024_1399_MOESM2_ESM.docx]

**Additional file 2 Sequences used in the present study**

| Accession No.^*^ | Name | Host order | Host species | Location  (country, province/city) | Collection date |
| --- | --- | --- | --- | --- | --- |
| KJ950906 | NrRV/NYC-A15 | *Rodentia* | *Rattus norvegicus* | United States, New York | 2013.06 |
| JF973686 | Rosavirus M-7 | *Rodentia* | *Peromyscus crinitus* | United States, California | 2010 |
| KJ158169 | Rosavirus 2 | *Human* | *Homo sapiens* | Gambia | 2008.10 |
| KX783421 | RNCW1002091R | *Rodentia* | *Rattus norvegicus* | China, Hong Kong | 2009.02 |
| KX783422 | RNYL1109081R | *Rodentia* | *Rattus norvegicus* | China, Hong Kong | 2008.09 |
| KX783423 | RNCW0602091R | *Rodentia* | *Rattus norvegicus* | China, Hong Kong | 2009.02 |
| KX783424 | RASK8F | *Rodentia* | *Rattus andamanensis* | China, Hong Kong | 2008.11 |
| KX783425 | RATLC11A | *Rodentia* | *Rattus andamanensis* | China, Hong Kong | 2009.03 |
| KX783426 | RAWKT4F | *Rodentia* | *Rattus andamanensis* | China, Hong Kong | 2008.10 |
| KX783427 | NFWKT7F | *Rodentia* | *Niviventer fulvescens* | China, Hong Kong | 2008.10 |
| KX783428 | NFSM6F | *Rodentia* | *Niviventer fulvescens* | China, Hong Kong | 2008.11 |
| KX783429 | RRTPC2A | *Rodentia* | *Rattus rattus* | China, Hong Kong | 2009.11 |
| KX783430 | RRTPC4A | *Rodentia* | *Rattus rattus* | China, Hong Kong | 2009.11 |
| KX783431 | NCHN06IO | *Rodentia* | *Niviventer coxingi* | China, Hong Kong | 2010.01 |
| KX783432 | NCGX12IN | *Rodentia* | *Niviventer coxingi* | China, Hong Kong | 2010.01 |
| KX783433 | RASM14A | *Rodentia* | *Rattus andamanensis* | China, Hong Kong | 2010.08 |
| MN116648 | rat08/rRoB/HUN | *Rodentia* | *Rattus norvegicus* | Hungary | 2018 |
| KX156156 | rodent/RL/PicoV/FJ2015 | *Rodentia* | *Rattus losea* | China, Fujian | 2015 |
| **PQ045667** | **YY2** | *Rodentia* | *Rattus norvegicus* | China, Yiyang | 2017.09 |
| **PQ045668** | **YY4** | *Rodentia* | *Rattus norvegicus* | China, Yiyang | 2017.09 |
| **PQ045669** | **YY6** | *Rodentia* | *Rattus norvegicus* | China, Yiyang | 2017.09 |
| **PQ045670** | **YY10** | *Rodentia* | *Rattus tanezumi* | China, Yiyang | 2017.09 |
| **PQ045671** | **YY27** | *Rodentia* | *Rattus tanezumi* | China, Yiyang | 2017.09 |
| **PQ045672** | **SMU442** | *Soricidae* | *Suncus murinus* | China, Guangzhou | 2016.01 |

^*^Sequences identified in this study are highlighted in bold.
