# Supplementary material for: A panoramic view of the molecular epidemiology, evolution, and cross-species transmission of rosaviruses
Source: Vet Res. 2024 Nov 8;55:145. doi: 10.1186/s13567-024-01399-3 (PMC11545274; doi:10.1186/s13567-024-01399-3)
Supplement: Supplementary file 3 — Additional file 3. Marginal likelihoods estimated via molecular clock models and coalescent models. [file 13567_2024_1399_MOESM3_ESM.docx]

**Additional file 3 The marginal likelihoods estimated of molecular clock models and coalescent models**

| Model of rate variation | Coalescent tree prior | Log marginal likelihood | Rank |
| --- | --- | --- | --- |
| Strick clock | Constant size | -8888.73 | 2 |
| Strick clock | Exponential growth | -8893.04 | 3 |
| **Strick clock** | **Bayesian skyline** | **-8888.22** | **1** |
| Uncorrelated lognormal relaxed clock | Constant size | -8902.03 | 5 |
| Uncorrelated lognormal relaxed clock | Exponential growth | -8904.46 | 6 |
| Uncorrelated lognormal relaxed clock | Bayesian skyline | -8896.45 | 4 |

The best-fitting combination of molecular clock models and coalescent tree prior are indicated in bold font.
