# Supplementary figures and images for: A panoramic view of the molecular epidemiology, evolution, and cross-species transmission of rosaviruses
Source: Vet Res. 2024 Nov 8;55:145. doi: 10.1186/s13567-024-01399-3 (PMC11545274; doi:10.1186/s13567-024-01399-3)

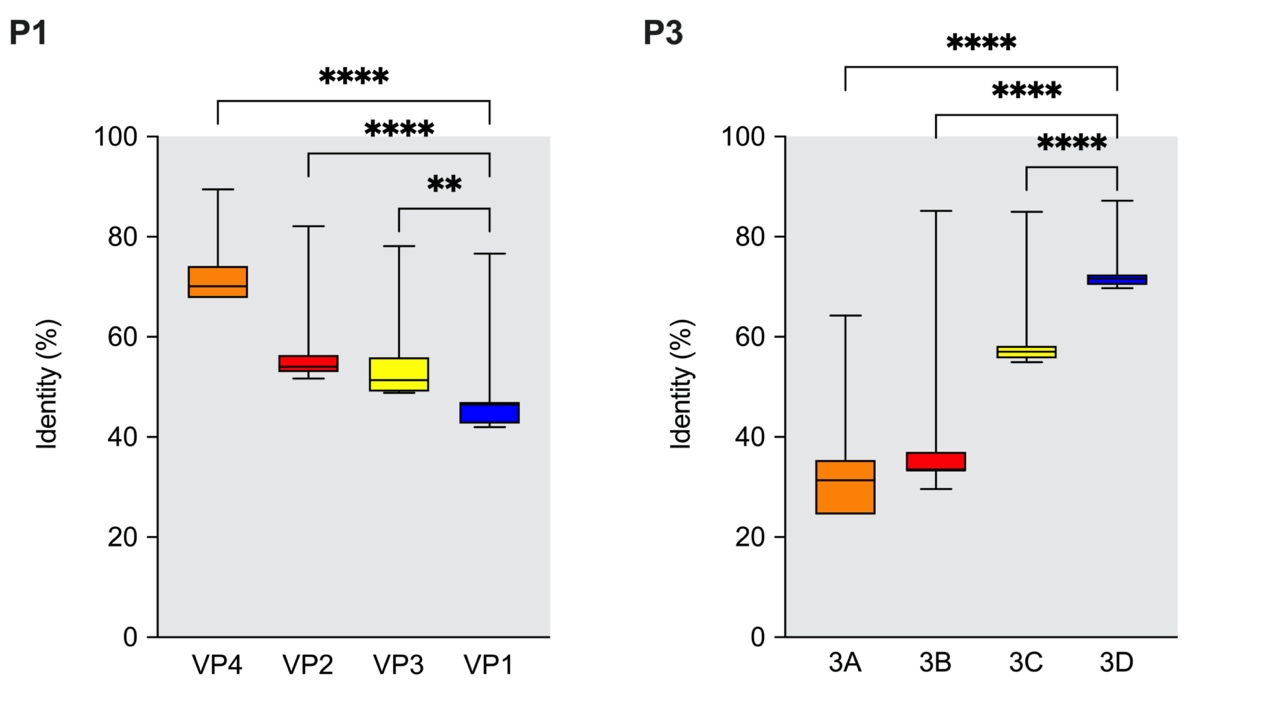

Supplement: Supplementary file 4 — Additional file 4. Mean pairwise amino acid identity of the P1 and P3 proteins of rosaviruses. **P < 0.01, ***P < 0.001, ****P < 0.0001. [file 13567_2024_1399_MOESM4_ESM.docx]
