# Supplementary material for: A panoramic view of the molecular epidemiology, evolution, and cross-species transmission of rosaviruses
Source: Vet Res. 2024 Nov 8;55:145. doi: 10.1186/s13567-024-01399-3 (PMC11545274; doi:10.1186/s13567-024-01399-3)
Supplement: Supplementary file 5 — Additional file 5. Migration paths of rosaviruses in seven geographical locations. [file 13567_2024_1399_MOESM5_ESM.docx]

**Additional file 5 Migration path of rosaviruses in seven geographical locations**

| FROM | TO | Mean migration rates | Mean indicators | BAYES FACTOR | POSTERIOR PROBABILITY |
| --- | --- | --- | --- | --- | --- |
| Fujian | Gambia | 0.998 | 0.184 | 0.718 | 0.120 |
| Fujian | Guangzhou | 1.012 | 0.202 | 0.718 | 0.120 |
| Fujian | Hong Kong | 0.954 | 0.225 | 2.293 | 0.303 |
| Fujian | Hungary | 1.008 | 0.216 | 71.384 | 0.931 |
| Fujian | United States | 1.042 | 0.202 | 24.430 | 0.822 |
| Fujian | Yiyang | 0.998 | 0.181 | 3.414 | 0.393 |
| Gambia | Guangzhou | 0.897 | 0.16 | 2.919 | 0.356 |
| Gambia | Hong Kong | 0.949 | 0.266 | 1.354 | 0.204 |
| Gambia | Hungary | 1.019 | 0.201 | 1.012 | 0.161 |
| Gambia | United States | 1.029 | 0.365 | 1.236 | 0.190 |
| Gambia | Yiyang | 1.011 | 0.225 | 1.643 | 0.238 |
| Gambia | Fujian | 0.894 | 0.181 | 1.872 | 0.262 |
| Guangzhou | Hong Kong | 1.008 | 0.222 | 1.613 | 0.234 |
| Guangzhou | Hungary | 0.919 | 0.236 | 1.020 | 0.162 |
| Guangzhou | United States | 0.939 | 0.216 | 1.096 | 0.172 |
| Guangzhou | Yiyang | 0.968 | 0.206 | 1.245 | 0.191 |
| Guangzhou | Fujian | 1.064 | 0.316 | 1.663 | 0.240 |
| Guangzhou | Gambia | 0.983 | 0.202 | 13.813 | 0.724 |
| Hong Kong | Hungary | 0.975 | 0.3 | 1.079 | 0.170 |
| Hong Kong | United States | 0.974 | 0.123 | 1.544 | 0.226 |
| Hong Kong | Yiyang | 0.991 | 0.393 | 3.883 | 0.424 |
| Hong Kong | Guangzhou | 1.013 | 0.932 | 1.236 | 0.190 |
| Hong Kong | Fujian | 1.017 | 0.818 | 4.008 | 0.432 |
| Hong Kong | Gambia | 0.98 | 0.116 | 1.633 | 0.236 |
| Hungary | Gambia | 1.009 | 0.234 | 1.281 | 0.195 |
| Hungary | Guangzhou | 1.011 | 0.161 | 1.400 | 0.210 |
| Hungary | Hong Kong | 1.052 | 0.423 | 1.663 | 0.240 |
| Hungary | United States | 0.992 | 0.192 | 2.245 | 0.299 |
| Hungary | Yiyang | 1.046 | 0.423 | 1.363 | 0.205 |
| Hungary | Fujian | 0.988 | 0.222 | 1.564 | 0.229 |
| United States | Gambia | 1.134 | 0.724 | 1.236 | 0.190 |
| United States | Guangzhou | 0.909 | 0.156 | 1.317 | 0.200 |
| United States | Hong Kong | 0.949 | 0.242 | 1.429 | 0.213 |
| United States | Hungary | 1.013 | 0.237 | 1.281 | 0.195 |
| United States | Yiyang | 0.975 | 0.188 | 1.148 | 0.179 |
| United States | Fujian | 0.96 | 0.172 | 1.524 | 0.224 |
| Yiyang | Gambia | 0.997 | 0.19 | 1.191 | 0.184 |
| Yiyang | Guangzhou | 0.964 | 0.136 | 116.593 | 0.957 |
| Yiyang | Hong Kong | 1.195 | 0.53 | 3.576 | 0.404 |
| Yiyang | Hungary | 1.007 | 0.393 | 0.842 | 0.138 |
| Yiyang | United States | 1.519 | 0.952 | 1.079 | 0.170 |
| Yiyang | Fujian | 0.962 | 0.154 | 5.934 | 0.529 |

Significant transmission routes with sufficient bayes factor (BF > 3) and posterior probability (PP > 0.5) support are marked with red font.
