# Supplementary material for: A panoramic view of the molecular epidemiology, evolution, and cross-species transmission of rosaviruses
Source: Vet Res. 2024 Nov 8;55:145. doi: 10.1186/s13567-024-01399-3 (PMC11545274; doi:10.1186/s13567-024-01399-3)
Supplement: Supplementary file 6 — Additional file 6. Cross-species transmission of rosaviruses in 10 host species [file 13567_2024_1399_MOESM6_ESM.docx]

**Additional file 6 Cross-species transmission of rosaviruses in 10 hosts species**

| FROM | TO | Mean migration rates | Mean indicators | BAYES FACTOR | POSTERIOR PROBABILITY |
| --- | --- | --- | --- | --- | --- |
| Peromyscus crinitus | Human | 1.017 | 0.581 | 1.347 | 0.140 |
| Peromyscus crinitus | Rattus norvegicus | 0.976 | 0.185 | 0.897 | 0.098 |
| Peromyscus crinitus | Rattus losea | 0.977 | 0.105 | 5.961 | 0.418 |
| Peromyscus crinitus | Suncus murinus | 1.002 | 0.08 | 0.785 | 0.087 |
| Peromyscus crinitus | Rattus andamanensis | 0.931 | 0.161 | 1.335 | 0.139 |
| Peromyscus crinitus | Niviventer fulvescens | 0.974 | 0.1 | 0.908 | 0.099 |
| Peromyscus crinitus | Rattus rattus | 1.028 | 0.112 | 1.762 | 0.175 |
| Peromyscus crinitus | Niviventer coxingi | 0.957 | 0.125 | 1.200 | 0.127 |
| Peromyscus crinitus | Rattus tanezumi | 0.97 | 0.108 | 0.730 | 0.081 |
| Human | Rattus norvegicus | 1.012 | 0.178 | 1.058 | 0.113 |
| Human | Rattus losea | 1.015 | 0.105 | 1.384 | 0.143 |
| Human | Suncus murinus | 0.939 | 0.084 | 0.942 | 0.102 |
| Human | Rattus andamanensis | 0.999 | 0.138 | 3.508 | 0.297 |
| Human | Niviventer fulvescens | 0.955 | 0.099 | 3.288 | 0.284 |
| Human | Peromyscus crinitus | 0.995 | 0.426 | 1.410 | 0.145 |
| Human | Rattus rattus | 1.05 | 0.124 | 2.273 | 0.215 |
| Human | Niviventer coxingi | 0.984 | 0.148 | 2.425 | 0.226 |
| Human | Rattus tanezumi | 0.955 | 0.087 | 0.818 | 0.090 |
| Rattus norvegicus | Rattus losea | 0.973 | 0.077 | 1.816 | 0.180 |
| Rattus norvegicus | Suncus murinus | 1.06 | 0.905 | 1.023 | 0.110 |
| Rattus norvegicus | Rattus andamanensis | 0.908 | 0.123 | 1.498 | 0.153 |
| Rattus norvegicus | Niviventer fulvescens | 1.014 | 0.045 | 1.775 | 0.176 |
| Rattus norvegicus | Rattus rattus | 0.95 | 0.08 | 2.185 | 0.209 |
| Rattus norvegicus | Niviventer coxingi | 0.905 | 0.083 | 2.258 | 0.214 |
| Rattus norvegicus | Rattus tanezumi | 1.063 | 0.993 | 0.919 | 0.100 |
| Rattus losea | Suncus murinus | 0.934 | 0.133 | 0.942 | 0.102 |
| Rattus losea | Rattus andamanensis | 1.012 | 0.277 | 1.576 | 0.160 |
| Rattus losea | Niviventer fulvescens | 0.963 | 0.112 | 1.058 | 0.113 |
| Rattus losea | Rattus rattus | 1.026 | 0.236 | 1.871 | 0.184 |
| Rattus losea | Niviventer coxingi | 0.986 | 0.294 | 1.046 | 0.112 |
| Rattus losea | Rattus tanezumi | 0.92 | 0.104 | 0.752 | 0.083 |
| Suncus murinus | Rattus andamanensis | 0.974 | 0.214 | 1.816 | 0.180 |
| Suncus murinus | Niviventer fulvescens | 1.011 | 0.129 | 1.615 | 0.163 |
| Suncus murinus | Rattus rattus | 1.035 | 0.162 | 2.440 | 0.228 |
| Suncus murinus | Niviventer coxingi | 0.977 | 0.17 | 1.486 | 0.152 |
| Suncus murinus | Rattus tanezumi | 0.935 | 0.148 | 1.997 | 0.194 |
| Rattus andamanensis | Niviventer fulvescens | 1.803 | 0.984 | 5.668 | 0.406 |
| Rattus andamanensis | Rattus rattus | 1.1 | 0.494 | 1.347 | 0.140 |
| Rattus andamanensis | Niviventer coxingi | 1.039 | 0.362 | 8.193 | 0.497 |
| Rattus andamanensis | Rattus tanezumi | 1.004 | 0.072 | 0.623 | 0.070 |
| Niviventer fulvescens | Rattus rattus | 1.057 | 0.217 | 2.185 | 0.209 |
| Niviventer fulvescens | Niviventer coxingi | 0.971 | 0.189 | 2.612 | 0.240 |
| Niviventer fulvescens | Rattus tanezumi | 0.976 | 0.109 | 1.249 | 0.131 |
| Niviventer fulvescens | Peromyscus crinitus | 1.045 | 0.189 | 1.969 | 0.192 |
| Niviventer fulvescens | Human | 0.964 | 0.185 | 3.508 | 0.297 |
| Niviventer fulvescens | Rattus norvegicus | 0.95 | 0.209 | 1.105 | 0.118 |
| Niviventer fulvescens | Rattus losea | 0.966 | 0.19 | 1.668 | 0.168 |
| Niviventer fulvescens | Suncus murinus | 0.987 | 0.101 | 0.988 | 0.107 |
| Niviventer fulvescens | Rattus andamanensis | 1.024 | 0.154 | 3.270 | 0.283 |
| Rattus rattus | Niviventer coxingi | 1.005 | 0.304 | 0.720 | 0.080 |
| Rattus rattus | Rattus tanezumi | 0.958 | 0.14 | 82.748 | 0.909 |
| Niviventer coxingi | Rattus tanezumi | 0.955 | 0.104 | 1.176 | 0.124 |
| Rattus norvegicus | Peromyscus crinitus | 0.996 | 0.092 | 1.871 | 0.184 |
| Rattus losea | Peromyscus crinitus | 1.027 | 0.165 | 11.104 | 0.573 |
| Suncus murinus | Peromyscus crinitus | 0.978 | 0.21 | 2.273 | 0.215 |
| Rattus andamanensis | Peromyscus crinitus | 0.998 | 0.116 | 1.069 | 0.114 |
| Rattus rattus | Peromyscus crinitus | 0.972 | 0.169 | 1.152 | 0.122 |
| Niviventer coxingi | Peromyscus crinitus | 0.965 | 0.142 | 2.097 | 0.202 |
| Rattus tanezumi | Peromyscus crinitus | 0.988 | 0.177 | 2.155 | 0.206 |
| Rattus norvegicus | Human | 0.925 | 0.116 | 1.913 | 0.188 |
| Rattus losea | Human | 0.997 | 0.196 | 1.224 | 0.129 |
| Suncus murinus | Human | 0.953 | 0.204 | 1.668 | 0.168 |
| Rattus andamanensis | Human | 1.017 | 0.113 | 4.607 | 0.357 |
| Rattus rattus | Human | 0.953 | 0.196 | 0.785 | 0.087 |
| Niviventer coxingi | Human | 0.988 | 0.141 | 3.583 | 0.302 |
| Rattus tanezumi | Human | 0.945 | 0.217 | 1.775 | 0.176 |
| Rattus losea | Rattus norvegicus | 0.936 | 0.208 | 0.977 | 0.105 |
| Suncus murinus | Rattus norvegicus | 0.97 | 0.164 | 1.498 | 0.153 |
| Rattus andamanensis | Rattus norvegicus | 0.936 | 0.142 | 524.908 | 0.984 |
| Rattus rattus | Rattus norvegicus | 1.029 | 0.194 | 0.385 | 0.044 |
| Niviventer coxingi | Rattus norvegicus | 0.985 | 0.212 | 1.359 | 0.141 |
| Rattus tanezumi | Rattus norvegicus | 1.05 | 0.246 | 1.273 | 0.133 |
| Suncus murinus | Rattus losea | 0.942 | 0.146 | 1.735 | 0.173 |
| Rattus andamanensis | Rattus losea | 1.092 | 0.415 | 1.093 | 0.117 |
| Rattus rattus | Rattus losea | 1.067 | 0.309 | 0.919 | 0.100 |
| Niviventer coxingi | Rattus losea | 0.97 | 0.285 | 1.708 | 0.171 |
| Rattus tanezumi | Rattus losea | 1.008 | 0.156 | 2.097 | 0.202 |
| Rattus andamanensis | Suncus murinus | 1.018 | 0.069 | 0.591 | 0.067 |
| Rattus rattus | Suncus murinus | 0.977 | 0.12 | 1235.832 | 0.993 |
| Niviventer coxingi | Suncus murinus | 1.025 | 0.091 | 1.285 | 0.134 |
| Rattus tanezumi | Suncus murinus | 1.097 | 0.196 | 1.435 | 0.148 |
| Rattus rattus | Rattus andamanensis | 1.111 | 0.342 | 1.116 | 0.119 |
| Niviventer coxingi | Rattus andamanensis | 1.05 | 0.306 | 4.282 | 0.341 |
| Rattus tanezumi | Rattus andamanensis | 1.019 | 0.182 | 2.318 | 0.219 |
| Rattus rattus | Niviventer fulvescens | 0.996 | 0.149 | 0.720 | 0.080 |
| Niviventer coxingi | Niviventer fulvescens | 1.031 | 0.112 | 3.833 | 0.316 |
| Rattus tanezumi | Niviventer fulvescens | 1.011 | 0.153 | 1.486 | 0.152 |
| Niviventer coxingi | Rattus rattus | 0.997 | 0.233 | 2.083 | 0.201 |
| Rattus tanezumi | Rattus rattus | 0.985 | 0.149 | 1.589 | 0.161 |
| Rattus tanezumi | Niviventer coxingi | 1.035 | 0.165 | 1.563 | 0.159 |

Significant transmission routes with sufficient bayes factor (BF > 3) and posterior probability (PP > 0.5) support are marked with red font.
